# Recent advances in volumetric super-resolution imaging through computational adaptive optics

**DOI:** 10.1186/s13041-026-01334-x

**Published:** 2026-07-28

**Authors:** Sangjun Park, Seungwon Jeong, Changhyun Yoo, Moonseok Kim

**Affiliations:** 1 Department of Medical Life Sciences, College of Medicine, The Catholic University of Korea, Seoul, 06591 Korea; 2 Department of Medical Sciences, Graduate School of The Catholic University of Korea, Seoul, 06591 Korea; 3 CMC Institute for Basic Medical Science, the Catholic Medical Center of The Catholic University of Korea, Seoul, 06591 Korea

**Keywords:** Adaptive optics, Super-resolution microscopy, Volumetric imaging, Deep-learning, Computational imaging

## Abstract

Optical microscopy’s power to resolve nanoscale structures deep within living tissues is limited by light diffraction and sample-induced aberrations. While super-resolution microscopy (SRM) has broken through the diffraction limit, its performance rapidly deteriorates in thick, heterogeneous specimens. Adaptive optics (AO) has emerged as a suitable solution to this challenge. By measuring and correcting wavefront distortions, AO restores imaging conditions, enabling SRM to achieve its full potential deep within complex biological samples like living brain tissue and developing organoids. The integration of AO with SRM (AO-SRM) is unlocking volumetric imaging capabilities, enhancing imaging depth and restoring nanoscale resolution. A recent advance has been the fusion of these hardware corrections with deep learning (DL). This convergence encompasses a dual paradigm of high-fidelity algorithmic restoration to bridge signal-to-noise ratio (SNR) gaps and systemic autonomy through self-driving frameworks for real-time hardware loop optimization. By employing intelligent agents that autonomously navigate aberration landscapes and trigger acquisition during biological events, this synergy maximizes experimental throughput while optimizing the photon budget to prevent sample degradation. DL algorithms enhance imaging capabilities by providing high-speed aberration prediction and robust computational reconstruction. This synergy not only boosts image contrast and resolution but also improves data processing efficiency and mitigates phototoxicity, enabling volumetric imaging with nanoscale precision. This review synthesizes recent developments in AO-integrated super-resolution imaging - encompassing image restoration approaches such as denoising and deblurring as well as wavefront correction techniques - and addresses how this convergence empowers the study of neuronal morphology and subcellular-scale dynamics deep within biological tissues.

## Introduction

**Fig. 1:**
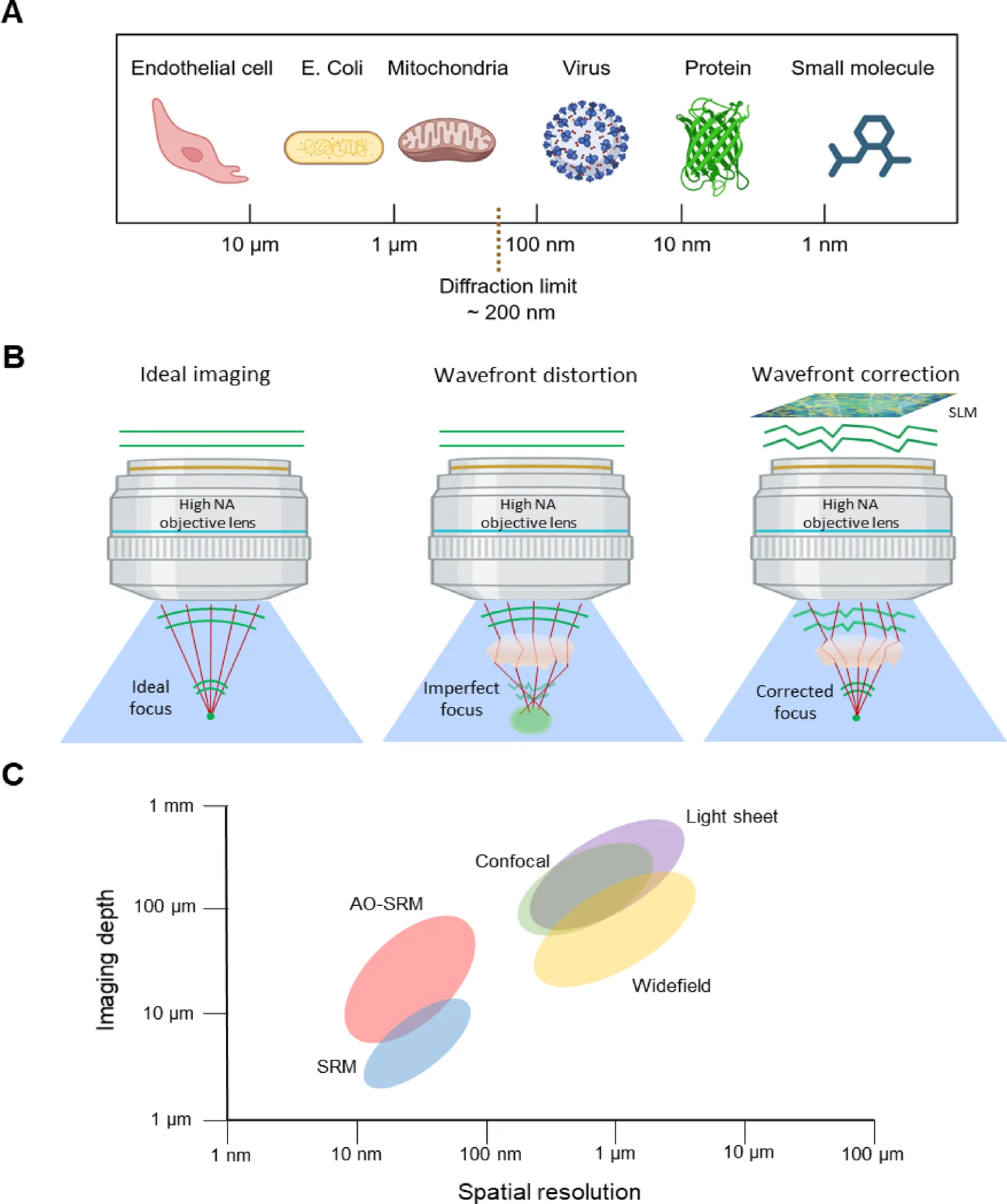
Super-resolution imaging integrated by AO. (A) Breaking the diffraction limit with super-resolution microscopy. Due to the diffraction limit, the viruses, proteins, and small molecules are unresolvable with conventional optical microscopy. (B) AO technique compensates wave distortion due to sample-induced aberrations for deep tissue imaging. (C) Trade-off relationship between spatial resolution and imaging depth in optical microscopies. The integration of AO with SRM (AO-SRM) unlocks nanoscale imaging in complex biological systems [32, 34]

Optical microscopy has served as the cornerstone of biological studies, offering an indispensable window into the structure and function of cells, tissues, and organs [1]. However, progress toward understanding the subcellular environment was long impeded by a physical constraint: the diffraction of light. The emergence of SRM redefined the boundaries of biological imaging by circumventing this long-standing diffraction limit. The spatial resolution of conventional optical microscopy is constrained by the diffraction of light, a physical principle described by Ernst Abbe’s criterion:

$$\:\mathrm{d}=\frac{\lambda\:}{2n\mathrm{s}\mathrm{i}\mathrm{n}{\uptheta\:}}=\frac{\lambda\:}{2\mathrm{N}\mathrm{A}}$$

where d is the minimum resolvable distance, λ is the excitation wavelength of light, n is the refractive index, θ is the half-angle of converging to a spot, and NA is the numerical aperture of the objective lens. For visible light, this restricts optical resolution to approximately 200 nm. This diffraction limit long rendered critical biological structures - such as individual viruses, protein complexes, and synaptic vesicles - unresolvable, leaving our understanding reliant on static electron micrographs or indirect biochemical assays as shown in Fig. 1(A) [2].

The advent of SRM provides a means to circumvent this long-standing physical barrier. By achieving resolutions at the tens-of-nanometers scale, these techniques bridged the gap between conventional optical microscopy and electron microscopy, granting an ability to visualize the organization and dynamic interactions of molecules in living systems. This technique has catalyzed studies across the biological sciences, enabling researchers to move beyond schematic diagrams to direct observation. Several principal strategies have emerged to achieve this feat, notably structured illumination microscopy (SIM), single-molecule localization microscopy (SMLM), and stimulated emission depletion (STED) microscopy.

First, SIM enhances spatial resolution based on the analysis of Moiré interference in the Fourier domain. SIM records multiple raw images with structured illumination patterns such as phase- and orientation-shifted sinusoidal stripes each respectively. Raw images containing Moiré fringes due to the incident pattern are Fourier transformed to extract otherwise inaccessible high-frequency structural detail of the sample. The super-resolved image is reconstructed through the synthesis of the shifted high-frequency information of all images in the Fourier plane, and the resulting maximum spatial frequency is the sum of the conventional cutoff frequency and the illumination frequency. When the illumination pattern frequency approaches the conventional cutoff frequency, it yields a two-fold improvement in lateral spatial resolution (up to 100 nm), compared to wide-field microscopy. Because only a few images are required, it has become a crucial tool for live-cell imaging with super-resolution maintaining high imaging speed and low phototoxicity [3–9].

Second, SMLM employs selective activation and on/off modulation of fluorescent molecules to temporally separate overlapping emission events. Such temporal sparsity enables reconstruction of molecular positions with nanometer accuracy. This approach relies on the stochastic activation of only a small subset of fluorophores at any given time. It enables the accurate localization of single molecule even if the distance between each molecule is closer than the diffraction limit.

In SMLM, the resolution is determined by the localization precision, which quantifies the statistical uncertainty in determining the center of an individual fluorescent emitter. This precision is primarily limited by two sources of noise: photon shot noise (from the inherent statistical fluctuations in photon detection) and background noise (autofluorescence, stray light, and detector noise). The theoretical localization precision can be expressed as follows:

$$ \left\langle {\left( {\Delta x} \right)} \right\rangle ^{2} = \frac{{s^{2} + a^{2} /12}}{N} + \frac{{4\sqrt \pi s^{3} b^{2} }}{{aN^{2} }} $$

where s is the standard deviation of point spread function (PSF), a is the effective pixel size of the detector, N is the total number of photons detected from the emitter, and b is the background noise in photons per pixel. This expression delineates two primary contributions to localization uncertainty. The first term, dominated by photon shot noise, quantifies the error arising from the statistical nature of photon emission and detection. The second term accounts for the error introduced by background noise, which becomes increasingly in low SNR. This equation reveals that localization precision is dependent on the number of collected photons (N). The uncertainty arising from photon shot noise diminishes as 1⁄√N, whereas the impact of background noise decreases more rapidly, as 1/N [10]. Representative implementations of the SMLM include photo-activated localization microscopy (PALM) [11–14], stochastic optical reconstruction microscopy (STORM) [15–19], and DNA point accumulation for imaging in nanoscale topography (DNA-PAINT) [20–24]. These techniques can achieve lateral resolutions of 10–20 nm and axial resolutions of 20–50 nm.

Third, STED microscopy is a point-scanning technique that achieves sub-diffraction resolution by superimposing a donut-shaped depletion beam onto a diffraction-limited excitation focus. It restricts fluorescence emission to a sub-diffraction central region by the use of two synchronized lasers. In STED microscopy, fluorophores are excited by a primary beam while a red-shifted beam selectively suppresses fluorescence in the periphery, effectively restricting fluorescence to the focal center. This depletion mechanism reduces the effective PSF with increasing beam intensity, and the corresponding resolution of STED can be expressed as

$${\mathrm{d}}_{\mathrm{S}\mathrm{T}\mathrm{E}\mathrm{D}}=\frac{{\uplambda}}{\mathrm{N}\mathrm{A}\sqrt{(1+{\mathrm{I}}_{\mathrm{S}\mathrm{T}\mathrm{E}\mathrm{D}}/{\mathrm{I}}_{\mathrm{s}\mathrm{a}\mathrm{t}})}}.$$

where $\:{\mathrm{I}}_{\mathrm{S}\mathrm{T}\mathrm{E}\mathrm{D}}$ is the intensity of the depletion beam and $\:{\mathrm{I}}_{\mathrm{s}\mathrm{a}\mathrm{t}}$ is the saturation intensity of the fluorophore for stimulated emission [25, 26]. Accordingly, the spatial resolution improves with high intensity of the depletion beam, and it allows resolutions of approximately 20–50 nm when the effective fluorescence spot becomes extremely small with higher the depletion beam intensity. In practice, the enhancement of resolution saturates due to the photobleaching, imperfect depletion, and chromatic aberration [27–31].

Despite this leap, the potential of SRM remained confined to thin, relatively transparent specimens. When applied to physiologically relevant contexts, such as deep within living animals, its power diminishes dramatically. The optical heterogeneity of biological samples induces sample-induced wavefront aberrations, which distort the PSF - the building block of a microscopic image. These aberrations reduce contrast and SNR, and in many cases, decrease the resolution afforded by SRM techniques.

To surmount this challenge, the field has turned to AO, a technology originally developed for astronomy to correct for atmospheric distortions [32]. In optical microscopy, depth-dependent aberrations severely distort the wavefront of incident beam and result in degradation of the focusing and image contrast in thick biological tissue. AO compensates sample-induced aberrations with the modulation of wavefront of incident light for deep tissue imaging, therefore it restores a diffraction-limited focus deep within biological tissues (Fig. 1B).

AO-SRM has proven to be a transformative synergy, unlocking the potential for nanoscale imaging in complex, intact biological systems. This leads the improvement of imaging depth of SRM, while maintaining super-resolution in Fig. 1C. Furthermore, the enhancement in photon efficiency mitigates the phototoxicity and photobleaching that can limit long-term live-cell studies [33].

In parallel with these hardware advances, computational approaches - especially DL - are further accelerating progress. Conventional computational approaches like deconvolution have long served to enhance image quality. DL is emerging as a transformative tool, enabling advanced image restoration, converting low-quality inputs into super-resolved outputs, and even powering digital AO to correct aberrations through computation. These DL-driven strategies offer high-speed aberration estimation that reduces the need for multiple hardware iterations, helping lower the light dose during sustained imaging.

This review addresses recent advances in volumetric imaging enabled by AO-SRM. While SRM has faced limitations in three-dimensional applications due to scattering and aberrations, its combination with techniques like light sheet microscopy (LSM) and AO has expanded its applicability for deep tissue imaging. Here, we focus on the implementation of SIM, SMLM, and STED with AO (AO-SIM, AO-SMLM, and AO-STED) and computational methods for volumetric biomedical imaging. In addition to wavefront correction methods, this review covers DL and computational adaptive optics approaches, embracing image restoration via denoising and deblurring. The specific methodologies are detailed in the following section, unified framework of adaptive optics in microscopy.

## Unified framework of adaptive optics in microscopy

**Fig. 2:**
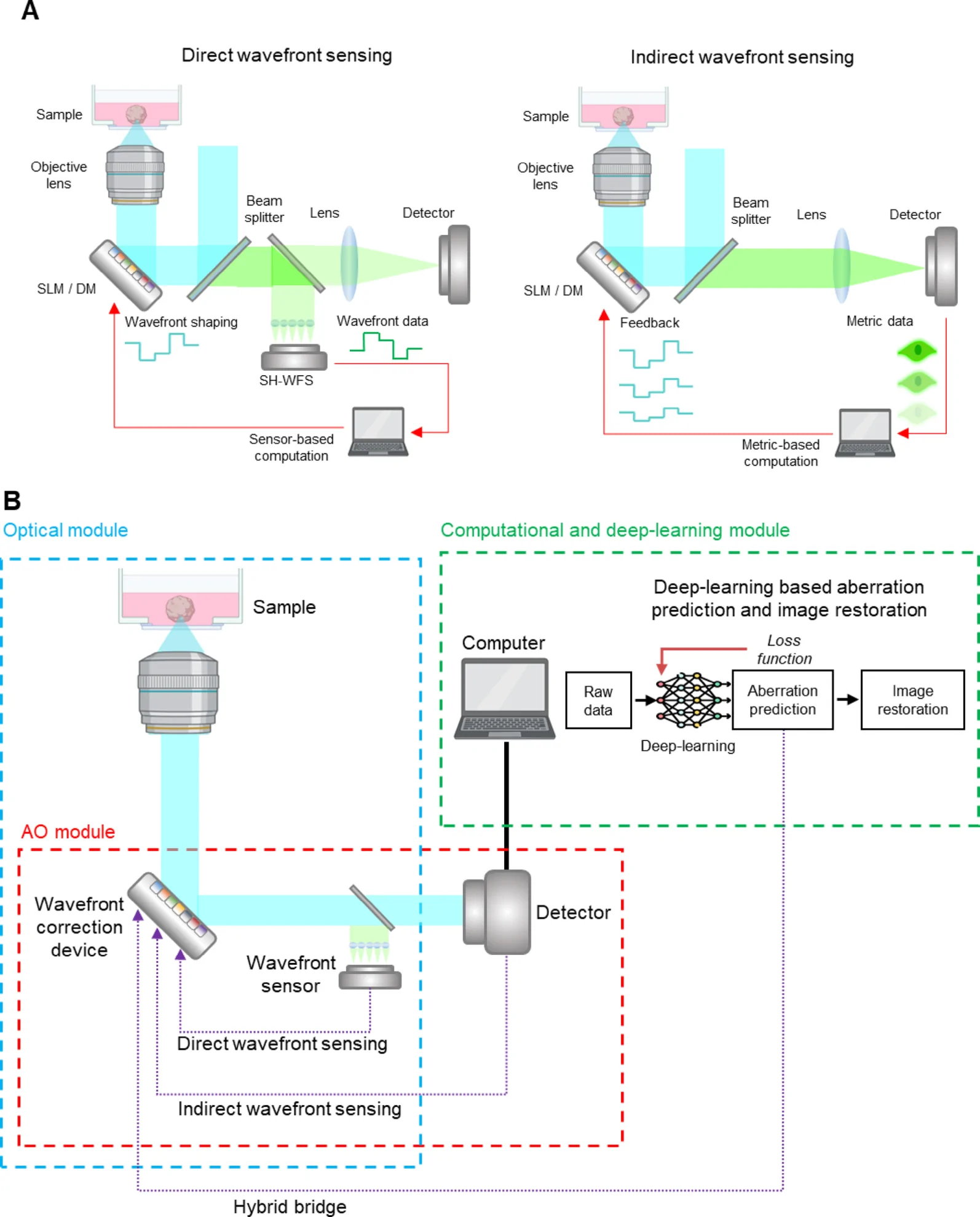
Principle of AO. **A** Detailed hardware AO architectures for Direct and Indirect Wavefront Sensing. Direct Wavefront Sensing (Left): Utilizes a dedicated SH-WFS to measure the phase distortion of the light beam directly. The sensor-based computation then provides high-speed, deterministic control signals to the SLM/DM for wavefront shaping. Indirect Wavefront Sensing (Right): Operates without a dedicated wavefront sensor. Instead, it relies on an iterative optimization process where the detector captures images, and a metric-based computation evaluates image quality (e.g., brightness, contrast, or sharpness) to provide feedback for sequential phase modulation by the SLM/DM. **B** Unified framework for AO-SRM. The framework integrates three synergistic modules to overcome sample-induced aberrations. (1) Optical Module: Represents the physical light path where sample-induced aberrations are introduced and subsequently corrected by the wavefront correction device. (2) AO Module: Illustrates the closed-loop control strategies, including direct wavefront sensing via a SH-WFS and Indirect wavefront sensing (sensorless) based on image quality metrics. (3) Computational and deep-learning Module: Facilitates both post-processing (DL-driven image restoration) and real-time hardware control. The hybrid bridge is a pathway where deep-learning models predict wavefront errors from raw data to provide rapid feedback to the wavefront correction device, bridging the gap between computational inference and physical correction

The AO implementation strategies are categorized as either direct sensing - using a physical sensor - or indirect sensing, which infers the aberration from image data as shown in Fig. 2A [35–39]. Direct wavefront sensing relies on a dedicated wavefront sensor such as Shack-Hartmann wavefront sensor (SH-WFS) to quantitatively measure the phase of light originating from a point-like source or guide star within the sample. The direct measurement of aberration enables correction at kilohertz rates, making it ideal for adapting to the rapid dynamics of in vivo imaging. However, its application in biology faces several hurdles: the scarcity of natural guide stars, the invasiveness of introducing artificial ones (e.g., fluorescent beads), signal degradation in scattering media, and the photon loss associated with splitting the fluorescence signal to the sensor.

The common type of wavefront sensor is the SH-WFS, which employs a microlens array to partition the incoming wavefront into numerous sub-apertures, each creating a focal spot on a camera. An ideal, planar wavefront produces a regular grid of spots; aberrations cause lateral displacements of these spots, which are proportional to the local slope of the wavefront. From this field of slopes, the overall phase aberration can be reconstructed using algorithms like least-squares fitting or Zernike polynomial. It provides a direct, non-interferometric measurement of the entire wavefront slope in a single shot, making it suitable for real-time correction in AO.

On the other hand, indirect wavefront sensing circumvents the need for a guide star and a dedicated sensor by iteratively optimizing an image quality metric. In this approach, a wavefront shaping device applies a series of predefined trial aberrations, and a corresponding metric - such as intensity, contrast, or sharpness - is calculated from the resulting image for each trial. An optimization algorithm, such as a hill-climbing or genetic algorithm, then searches for the aberration correction that maximizes the metric. While this indirect approach offers advantages in hardware simplicity and is applicable even in opaque tissues, its iterative process is slow, often taking seconds to minutes to converge. This limits its utility for imaging dynamic processes and can increase the risk of phototoxicity due to the need for multiple image acquisition. To reduce this measurement overhead, phase diversity (PD) has emerged as a powerful sensorless approach that infers wavefront aberrations from a limited set of images with known phase modulations, which was subsequently extended to multiplane formulations for three-dimensional widefield fluorescence microscopy [40]. Furthermore, recent technical advancements have accelerated this process, establishing a highly sensitive PD wavefront sensing scheme on approximately 100 ms scale that achieves a hundredfold speedup in corrective element calibration and restores diffraction-limited resolution without supplementary hardware [41]. To further overcome the latency and phototoxicity constraints, there is a breakthrough technology toward computational wavefront sensing framework. Recently, DL models bypass the need for iterative trials by directly inferring the required phase compensation from a single or a few intensity images.

The next step after wavefront sensing is the modulation of incident beam pattern with a wavefront shaping device to compensate the wavefront distortion from sample-induced aberration. The widely used devices are deformable mirrors (DMs) or spatial light modulators (SLMs). DMs are reflective surfaces whose shape is controlled by an array of underlying actuators. By applying voltages, the mirror surface can be deformed to produce a shape that is conjugate to the measured aberration. DMs offer rapid response times suitable for real-time correction, are independent of polarization, and operate effectively across broad spectral ranges, making them compatible with the polychromatic fluorescence emission common in biological imaging. SLMs are liquid-crystal-based pixel arrays that modulate the phase of light. For instance, early widefield fluorescence microscopy frameworks utilized large-throw DMs to rapidly compensate for depth-dependent spherical aberrations and perform rapid, motionless axial focusing without mechanical sample disturbance [42]. Offering a number of control elements than DMs, SLMs can generate complex and precise phase patterns, making them ideal for correcting high-order aberrations. However, they are slower than DMs and are restricted to specific polarization states. The phase modulation is wavelength-dependent, which can introduce chromatic inaccuracy in the use of broadband light sources.

Recently, the integration of DL into SRM (DL-SRM), as a computational wavefront correction or image restoration framework involving denoising and deblurring, has evolved along two distinct but complementary paths: the refinement of image fidelity through post-acquisition algorithms and the realization of systemic autonomy through real-time hardware control. Within the scope of image refinement, DL serves as a sophisticated algorithmic framework to mitigate intrinsic degradations in fluorescence imaging, notably phototoxicity and low SNR [43]. There has been a discernible shift from conventional inverse modeling toward advanced data-driven architectures—such as Content-Aware Image Restoration (CARE) [44], and Residual Channel Attention Networks (RCAN) [45]—to enhance spatial resolution and recover biological details obscured by diffraction limits and noise. By establishing a systematic integration of denoising [46–51], deconvolution [52–54], and restoration protocols [55–60], it becomes evident how computational restoration bridges the gap between degraded raw acquisitions and high-fidelity scientific data.

In contrast, recent frameworks advocate for systemic autonomy via autonomous microscopy, integrating DL as an active component of the hardware control loop [61]. This transition decouples SR from conventional post-processing, instead utilizing DL for dynamic decision-making. By determining the what (phenotype identification), when (predictive event triggering), and how (reinforcement learning-based optimization) of imaging, this strategy enhances efficiency. It minimizes sample damage by restricting high-intensity illumination to the biologically relevant regions and moments.

The convergence of DL paradigms is particularly transformative for AO-SRM. To computationally recover a clear image, their success depends on a stable and well-characterized PSF, which is often distorted by sample-induced aberrations in deep tissue. AO serves as the essential hardware-level solution to these aberrations; however, the complexity of estimating phase distortions often presents a bottleneck in live-cell SR imaging. This evolution represents a functional integration of sensing modalities; DL-driven wavefront estimation achieves the high-speed performance of direct sensing while maintaining the hardware simplicity of indirect, sensorless approaches. By learning the complex mapping between PSFs and their underlying phase distortions, these models effectively turn the imaging camera into a virtual wavefront sensor. The DL agents are now capable of automating aberration estimation and focus correction, enabling AO systems to perform deep-tissue SR imaging in highly scattering environments that hinder classical Fourier-based estimation methods. By combining AO’s ability to physically recover the PSF with the algorithmic fidelity of restoration and the autonomous decision-making agents, researchers can achieve a triple synergy: AO physically restores signal integrity, DL algorithms maximize final image resolution, and autonomous controllers ensure that these high-precision tools are deployed with optimal efficiency and minimal photodamage.

As illustrated in the unified framework in Fig. 2B, AO provides a robust system to counteract these distortions through the integrated synergy of an optical module, AO module, and computational and deep-learning module. Within this architecture, wavefront shaping devices such as DMs or SLMs physically compensate for sample-induced aberrations by applying a conjugate phase pattern to restore signal integrity such as hybrid bridge. This physical correction is increasingly governed by a dual-path intelligence layer that facilitates both systemic autonomy and algorithmic fidelity. In this unified architecture, the traditional distinction between hardware-based and image-based sensing is blurred. The DL module acts as a unified inference engine that processes raw data to simultaneously drive the AO module for real-time hardware correction and the computational module for super-resolved restoration. According to the self-driving paradigm, reinforcement learning agents and event-triggered neural networks act as real-time controllers within the hardware loop to autonomously navigate complex aberration landscapes and predict necessary corrections in real time. Simultaneously, high-fidelity restoration architectures such as RCAN and CARE bridge SNR gaps through post-acquisition or learned-prior reconstruction, ensuring that nanoscale resolution is maintained even in photon-sparse or scattering environments. The convergence of these hardware corrections and DL strategies extends the practical capabilities of SRM by enabling rapid aberration prediction and image restoration. This integrated approach effectively stabilizes the PSF within complex biological specimens such as brain tissue and organoids while mitigating phototoxicity through efficient, event-driven acquisition schemes. The unified DL-AO framework transforms conventional microscopy into a DL-driven platform capable of long-term volumetric mapping of dynamic biological processes at the nanoscale.

### AO-SIM: restoring fidelity for deep and dynamic imaging

SIM is a widely accessible method for doubling the resolution of widefield microscopy. However, its reliance on the precise projection and detection of patterned light makes it sensitive to aberrations. For deep tissue imaging, sample-induced aberrations blur the illumination patterns, leading to a loss of modulation contrast. This degradation not only reduces the SNR of high-frequency information but also distorts the optical transfer function, resulting in reconstruction artifacts that can obscure or even create false structures.

The integration of AO provides a direct and robust solution to this challenge. By correcting wavefront distortions, AO-SIM maintains high-contrast illumination patterns deep within sample, thereby preserving the raw data integrity required for accurate reconstruction. This capability has been foundational in moving SIM from an application restricted to cultured cells to a useful tool for in vivo biology.

**Fig. 3:**
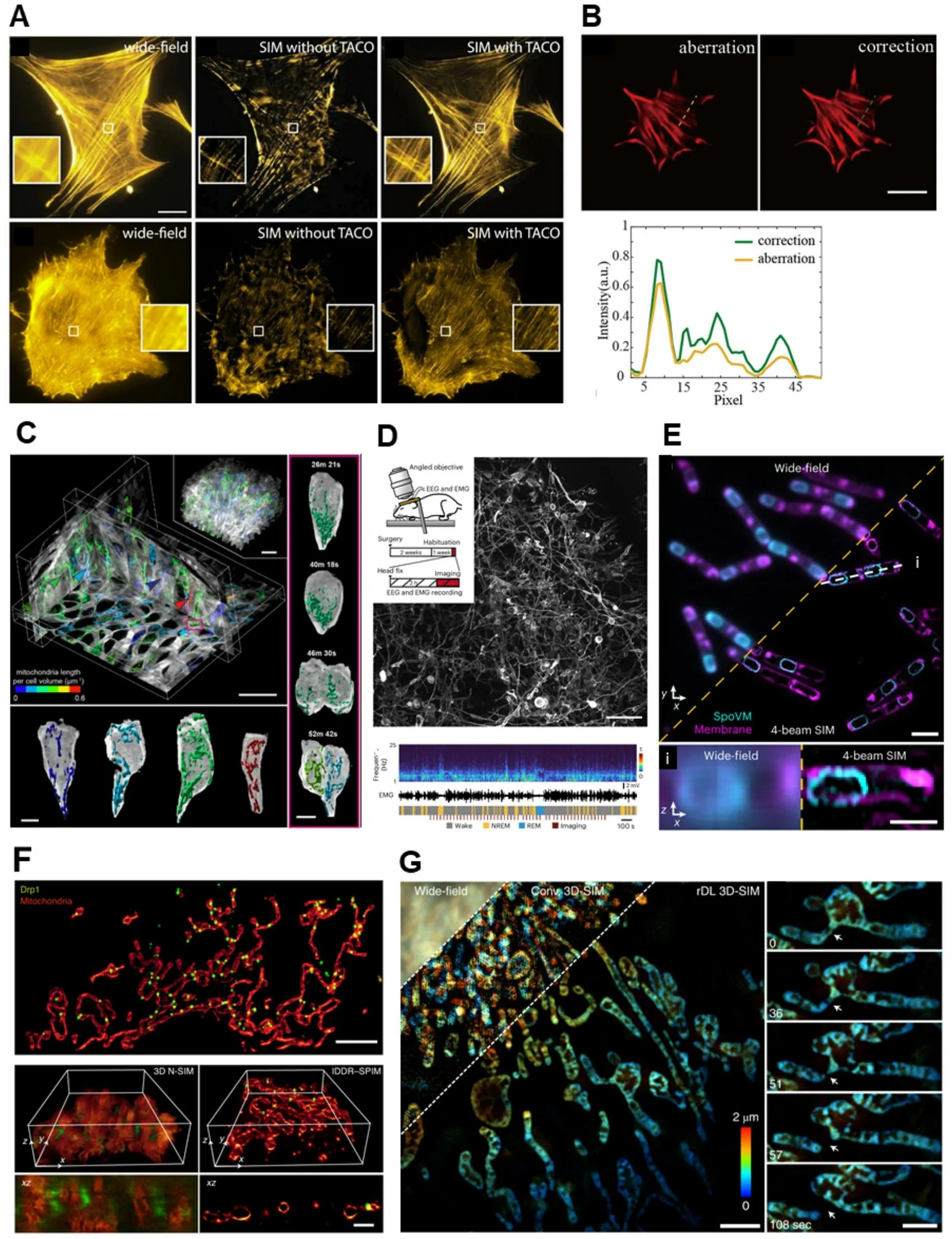
AO-SIM. **A** SIM imaging with the TACO system corrects aberrations to enhance the resolution and accuracy of super-resolution images. Scale bar: 20 μm. Adapted from Gong D, Scherer NF. Biomed Opt Express. 2023;14(12):6381–96 [62]. **B** With AO-SIM, super-resolution imaging results of actin filaments stained with Phalloidin in BHK cells. Scale bar: 20 μm. Adapted from Zheng Y, Chen J, Wu C, Gong W, Si K. Cytometry Part (A) 2021;99(6):622–31 [63]. (**C**) Applying AO to super-resolution imaging modes in living zebrafish enables precise 3D structures of mitochondria and cell membranes as well as dynamic cellular processes. Scale bar: 10 μm. Adapted from Fu T-M, Liu G, Milkie DE, Ruan X, Görlitz F, Shi Y, et al. Nat Methods. 2026;23(6):1184–95 [64]. (**D**) Long-term tracking of spinule numbers and morphological changes in dendritic spines and axonal boutons during mouse sleep-wake cycles using MLS-SIM. Scale bar: 10 μm. Adapted from Zhang Y, Bai L, Wang X, Zhao Y, Zhang T, Ye L, et al. Nat Methods. 2025;22(1):177–86 [65]. **E** Four-beam SIM improves axial resolution compared to conventional 3D SIM to visualize fine structures of *(B) Subtilis* at near-isotropic super-resolution. Scale bar: 2 μm (upper panel), 1 μm (lower panel). Adapted from Li X, Wu Y, Su Y, Rey-Suarez I, Matthaeus C, Updegrove TB, et al. Nat Biotechnol. 2023;41(9):1307–19 [66]. **F** Through dynamic 5-dimensional super-resolution imaging via IDDR–SPIM that Drp1 oligomers are involved in mitochondrial fission and branch formation. Scale bar: 5 μm (upper panel), 1 μm (lower panel). Adapted from Zhao Y, Zhang M, Zhang W, Zhou Y, Chen L, Liu Q, et al. Nat Methods. 2022;19(3):359–69 [67]. **G** rDL-driven 3D-SIM and LLS-SIM can track mitochondrial cristae remodeling and multilayered nucleolar condensation-disassembly processes under long-term conditions. Scale bar: 3 μm (left panel), 2 μm (right panels). Adapted from Qiao C, Li D, Liu Y, Zhang S, Liu K, Liu C, et al. Nat Biotechnol. 2023;41(3):367–77 [60]. **H** Bayesian deformable phase-space alignment (DPA) time-lapse image SR (TISR) enables reliable long-term super-resolution live-cell imaging by classifying mitochondria-peroxisome contacts. Scale bar: 3 μm (upper panel), 1 μm (lower panel). Adapted from Qiao C, Liu S, Wang Y, Xu W, Geng X, Jiang T, et al. Nat Biotechnol. 2025 [68]. **I** Meta-rLLS-VSIM achieves quasi-isotropic super-resolution (lateral 122 nm, axial 162 nm) for long-term multi-color 4D imaging in living COS-7 cells. Scale bar: 3 μm (left panel), 1 μm (right panel). Adapted from Qiao C, Li Z, Wang Z, Lin Y, Liu C, Zhang S, et al. Nat Methods. 2025;22(5):1059–69 [69]. **J** Long-term tracking of the mitochondrial cristae and nucleoids in living cells using DFCAN and DFGAN-based SISR techniques. Scale bar: 3 μm (upper panels), 1 μm (lower panels). Adapted from Qiao C, Li D, Guo Y, Liu C, Jiang T, Dai Q, et al. Nat Methods. 2021;18(2):194–202 [58].

The tandem aberration correction optics (TACO) framework addresses the challenge of spatially-varying aberrations in SIM by integrating two distinct AO stages. While conventional pupil AO employs a DM in the detection path to rectify global, system-induced aberrations, the TACO approach incorporates a SLM at a plane conjugate to the aberration source to pre-compensate for localized phase distortions in the illumination path. This dual-stage configuration ensures that high-fidelity sinusoidal patterns are projected onto the sample, overcoming the non-isoplanatic limitations that typically degrade super-resolution reconstruction in complex biological media. To eliminate the need for complex hardware-based wavefront sensors, TACO utilizes a sensorless, image-based sensing strategy governed by the stochastic parallel gradient descent (SPGD) algorithm. The system iteratively optimizes a specialized image quality metric—defined by the contrast ratio of diffraction orders in the Fourier domain—to estimate the necessary phase corrections. For the high-dimensional control of the SLM, computational efficiency is significantly enhanced through the application of a Gaussian kernel during updates, which smooths the phase topography to match physical aberrations and accelerates the convergence of the optimization process toward an ideal unaberrated state. The practical utility of the TACO system is demonstrated through the high-resolution imaging of actin networks in human fibroblast and melanoma cells, as exemplified in Fig. 3A. By actively recovering the integrity of the structured illumination pattern, the framework eliminates reconstruction artifacts and restores the optical sectioning capability previously hindered by sample inhomogeneity. Consequently, TACO enables the clear resolution of fine cellular structures, such as actin branches separated by as little as 155 nm, which remain indistinguishable under standard widefield or uncorrected SIM conditions. The effectiveness of AO-SIM is intrinsically linked to the strategic placement of correction elements within the optical architecture. In advanced configurations such as the TACO framework, a SLM is positioned in the illumination path to perform conjugate AO, pre-compensating for spatially varying distortions that would otherwise blur the excitation patterns. Simultaneously, a DM is integrated into the detection path for pupil-plane AO to rectify global, system-induced aberrations in the emitted fluorescence signal. This dual-pathway, online correction timing ensures that high-fidelity sinusoidal patterns are projected onto the sample in real-time, effectively restoring the modulation transfer function (MTF) and minimizing reconstruction artifacts that typically plague deep-tissue SIM [62].

Recent research presents a sensorless AO framework for SIM that utilizes a convolutional neural network (CNN) to mitigate optical aberrations in deep-tissue imaging. By establishing a direct mapping between distorted 307 × 307 pixel structured patterns and the coefficients of the 4th to 15th Zernike modes, the AlexNet-based model achieves rapid aberration sensing within approximately 10.1 ms. The algorithm demonstrates exceptional precision, reaching a correlation index of 0.9986 between the predicted and actual aberration phases, effectively replacing traditional hardware-based wavefront sensors while maintaining high computational efficiency. Wavefront correction is implemented by loading the predicted compensation phase onto a SLM, which restores the ideal structured illumination patterns distorted by refractive index mismatches in biological specimens. This correction algorithm is highly robust, maintaining effectiveness across various spatial periods and illumination conditions; notably, the post-correction correlation coefficient can improve by up to 2.57 times compared to the initial distorted state. The practical utility of the system is validated through high-resolution imaging of Phalloidin-labeled actin filaments in BHK cells in Fig. 3B, where the framework eliminates artifacts and significantly enhances subcellular structural definition [63].

The multimodal optical scope with adaptive imaging correction (MOSAIC) system addresses the constraints of conventional microscopy by integrating diverse modalities—including lattice light-sheet, structured illumination, and two-photon microscopy—into a single reconfigurable platform. A cornerstone of this architecture is a dual-pathway AO framework that employs direct wavefront sensing to independently measure and compensate for sample-induced aberrations in both the excitation and detection channels. This hardware-level correction, modulated via SLMs and DMs, restores diffraction-limited resolution and enhances SNR within deep, scattering multicellular environments. Complementing these hardware corrections, MOSAIC utilizes advanced computational algorithms, such as DL-driven CARE for high-fidelity denoising and a tiled reconstruction strategy to address spatially varying aberrations across large fields of view. These computational enhancements, integrated with the GPU-accelerated PetaKit5D pipeline, facilitate the real-time processing of massive datasets and enable quantitative 4D observations of subcellular dynamics. As demonstrated in Fig. 3C, the AO-LLS-SIM (Lattice Light-Sheet Structured Illumination Microscopy) mode achieves sufficient resolution to segment mitochondrial networks in developing zebrafish embryos, allowing for precise morphometric analysis and the tracking of organelle partitioning during cell division in vivo [64].

Multiplexed line-scanning SIM (MLS-SIM) represents a significant advancement in SRM by enabling high-fidelity 3D imaging in awake and behaving animals. Unlike conventional wide-field 3D-SIM, which requires multiple frames and is prone to motion artifacts, MLS-SIM achieves 3D super-resolution within a single continuous scan. The system utilizes multiplexed and patterned line excitations to reach a lateral resolution of 150 nm in its linear form and approximately 100 nm in its nonlinear saturated form. This high-speed architecture reduces the acquisition time for local structures to approximately 1 ms, providing inherent immunity to severe global sample motions. The framework’s resilience to physiological movement is powered by a specialized row-by-row image registration pipeline. Since MLS-SIM data is collected line by line, motion-induced image distortions are modeled as rigid translations of individual rows. The algorithm estimates these spatial shifts using 2D cross-correlation against a reference volume, followed by refinement through Gaussian kernel curve fitting to assume continuous sample motion. This computational approach preserves local super-resolution even in the presence of instantaneous animal movements exceeding 100 μm/s, allowing for the precise tracking of fine neurites and synaptic structures in awake states as shown in Fig. 3D. The core of the MLS-SIM reconstruction suite is a joint deconvolution algorithm that fuses information from raw image strips categorized into groups based on their effective PSFs. To enhance optical sectioning and reject out-of-focus background, the framework employs a multilayer reconstruction strategy that approximates the 3D sample as discrete 2D layers. This algorithmic efficiency enabled the longitudinal observation of fast morphological dynamics, such as spinule recurrence and the spatial reorganization of PSD-95 clusters, across sleep-wake cycles and over multiple days in the primary somatosensory cortex [65].

Recent study addresses the inherent anisotropy of three-dimensional SIM by implementing a hardware-based four-beam interference strategy that achieves near-isotropic approximately 160 nm axial resolution. To mitigate spatial frequency gaps and refractive index mismatches, the authors developed a robust bead-based aberration sensing and correction algorithm utilizing 100 nm fluorescent fiducial markers to characterize the system’s PSF. A closed-loop feedback mechanism calculates the axial displacement between the PSF offset and the interference pattern’s standing-wave maxima, actively driving a piezoelectric mirror and sample stage to maintain interferometric stability within a 10–20 nm tolerance. Furthermore, spherical aberrations are minimized through the use of RI-matched immersion media, such as 45.6% iodixanol, ensuring the high-contrast illumination necessary for accurate volumetric reconstruction. Complementing the hardware approach, a sophisticated computational pipeline utilizing multi-step DL is introduced to achieve a fully isotropic resolution of approximately 120 nm without additional hardware modification. This framework employs a six-direction isotropization model that learns to reverse anisotropic degradation by training on high-resolution lateral views, effectively restoring axial information obscured by diffraction. To facilitate prolonged 4D imaging, a dual-stage 3D RCAN is integrated for high-fidelity denoising, allowing for high-quality reconstructions from raw data acquired at low illumination intensities (0.5 W/cm^2^). This synergistic integration of active hardware alignment and DL-driven restoration provides a 2–3 fold enhancement in volumetric resolution compared to state-of-the-art lattice light-sheet or I^5^S implementations, enabling the detailed visualization of rapid organelle dynamics in live cells as shown in Fig. 3E [66].

As a complementary computational restoration approach rather than active AO, the Isotropic divide stages-to-process selective plane illumination microscopy (IDDR-SPIM) framework circumvents the limitations of traditional 3D super-resolution by integrating static wavefront engineering with a refined selective plane illumination design. Instead of employing active wavefront sensing, the system utilizes a custom-designed double-ring modulation plate placed at the back focal plane of the illumination objective to perform spatial filtration of the excitation beam. This passive wavefront modulation generates a 0.45-µm-thick Bessel-type light sheet while suppressing axial side lobes to 35%—a 1.7-fold reduction compared to standard lattice light sheets—thereby minimizing image artifacts and maximizing photon efficiency for sustained live-cell imaging. To address residual optical blurring and sensor pixelization, the researchers implemented the Isotropic Divide Stages-to-Process neural network, a divide-and-conquer computational strategy. This DL algorithm decouples complex image degradation into three sequential stages: denoising, deblurring, and depixelization. By incorporating a DRUNet-based pre-denoising adapter and a perceptual loss function alongside classic mean-square-error loss, the network optimizes structural fidelity and resolution beyond the diffraction limit. Furthermore, an axial-to-lateral self-supervised learning scheme is applied to the reconstructions, enabling the inference of approximately 100-nm isotropic resolution from a single noisy, diffraction-limited volumetric input. The synergy between optical wavefront control and DL computation yields superior spatiotemporal performance, achieving a volumetric imaging rate of up to 17 Hz with negligible photobleaching (less than 0.01% per volume). This capability allows for the precise 5D visualization of rapid intracellular events, such as Drp1-mediated mitochondrial division and branching, which were previously unresolvable due to axial blurring and insufficient temporal resolution. Moreover, the established isotropic divide model demonstrates robust generalization through cross-mode applications, effectively enhancing 3D data from other modalities, such as spinning disk confocal microscopy, to super-resolution levels without additional hardware modifications (Fig. 3F) [67].

The Rationalized DL (rDL) framework effectively mitigates the issues of spectral bias and reconstruction artifacts prevalent in conventional DL-SRM by integrating deterministic physical models into the neural architecture. Its three-branch design—comprising Primary Feature Extracting (PFE), Moiré Pattern Extracting (MPE), and Feature Coalescing and Denoising (FCD) modules—utilizes prior knowledge of structured illumination patterns and PSF to constrain network inferences within a physically plausible manifold. This integration ensures that the resulting denoised raw images are optimized for high-fidelity analytical reconstruction, providing a more quantifiable and faithful representation of subcellular structures than standard black-box models. To address the challenge of acquiring high-quality ground-truth data in dynamic specimens, the rDL scheme employs self-supervised computational strategies based on spatiotemporal continuity. Temporally Interleaved (TiS-rDL) and Spatially Interleaved (SiS-rDL) protocols leverage redundancy in high-speed, Nyquist-sampled datasets to achieve denoising outcomes equivalent to supervised training. These algorithmic advancements enable sustained volumetric imaging of rapid bioprocesses—such as ciliary kinetics at 684 fps and organelle inheritance during mitosis—under minimal light intensity, thereby significantly extending the viable imaging duration while minimizing phototoxicity. Furthermore, the rDL framework demonstrates significant resilience to optical aberrations, maintaining structural accuracy even under non-ideal wavefront conditions. Analytical evaluations show that the model remains robust against spherical and coma aberrations, with resolution degradation limited to less than 2.3% for wavefront errors as high as 0.4λ. This inherent tolerance to PSF distortions and illumination phase errors highlights rDL’s potential for synergy with AO, promising to facilitate minimally invasive 5D SR imaging in optically complex biological environments where conventional methods typically fail (Fig. 3G) [60].

The Deformable Phase-space Alignment Time-lapse Image Super-Resolution (DPA-TISR) framework addresses the inherent instability of single-image super-resolution (SISR) by implementing a recurrent network-based propagation (RNP) scheme to capture long-range temporal dependencies. By leveraging bidirectional feature gathering, the architecture ensures superior temporal consistency and noise robustness compared to sliding-window or attention-based models. This transition from static to time-lapse super-resolution (TISR) facilitates high-fidelity imaging of rapid biological dynamics, such as clathrin-mediated endocytosis and mitochondrial cristae transformations, without the artifacts typically associated with motion-induced discontinuities.Central to the model’s precision is the Deformable Phase-space Alignment (DPA) mechanism, which performs subpixel feature alignment within the frequential space. By applying Real-valued Fast Fourier Transform (RFFT) to extract phase residuals, the algorithm estimates motion and aligns features to compensate for complex intracellular movements that traditional spatial warping or optical flow methods often fail to resolve. This computational approach to wavefront correction functions as a digital adaptive optics layer, adaptively learning global and local phase shifts to maintain structural integrity even under the low-SNR conditions necessitated by long-term live-cell observation.To ensure scientific rigor in downstream analysis, the framework incorporates a Bayesian DL module to quantify both aleatoric and epistemic uncertainties. Through an iterative fine-tuning process designed to minimize the expected calibration error, the system generates reliable confidence maps that correspond strictly to true inference accuracy. This calibrated, error-aware imaging enabled the quantitative characterization of peroxisome-mediated mitochondrial fission over 10,000 timepoints, demonstrating the capacity of the model to support high-throughput biological discovery with verified fidelity (Fig. 3H) [68].

Conventional lattice light-sheet structured illumination microscopy (LLS-SIM) is fundamentally limited by single-orientation structured illumination, which results in anisotropic resolution and potential image distortions. To address this, the authors developed a Virtual Structured Illumination (VSI-SR) strategy that utilizes a deep neural network (DNN) to infer 1D super-resolution intermediates from diffraction-limited LLSM data. By computationally rotating the input data and applying the trained model, the framework generates anisotropic SR components across multiple orientations, which are then integrated using a generalized Wiener filter in Fourier space. This process achieves a laterally isotropic resolution of approximately 120 nm without requiring modifications to the core optical hardware.To mitigate the high data and temporal costs typical of standard DL models, the system implements a meta-learning-empowered adaptation scheme. Based on the model-agnostic meta-learning (MAML) algorithm, the framework trains a meta-model to capture transferable, structure-independent features of the SR task. This allows the model to adapt to novel biological specimens or varying SNRs in tens of seconds using minimal training data—a 720-fold reduction in training time compared to standard procedures. The architecture employs a multi-slice conditional generative adversarial network and a Fourier space-regularized loss function to preserve structural continuity along the axial axis and ensure high-fidelity volumetric reconstruction. Axial resolution, which is traditionally significantly lower than lateral resolution, is enhanced through a reflective light-sheet configuration that captures dual symmetrical views of the specimen. These views are fused by the Richardson-Lucy dual-cycle fusion network, a self-supervised model that integrates physical PSF priors directly into its architecture via a Richardson-Lucy deconvolution module. Rather than estimating sample-induced optical aberrations or performing adaptive wavefront correction, this physics-informed approach assumes the PSF as prior knowledge to resolve the ill-posedness of axial enhancement, resulting in a near-isotropic axial resolution of 160 nm. The resulting 4D imaging capability enables the high-speed, long-term observation of rapid subcellular bioprocesses and complex organelle interactomes (Fig. 3I) [69].

The BioSR dataset addresses the discrepancy between simulated DL-SRM performance and real-world microscopy. By providing well-matched image pairs across varying SNR and structural complexities, the study establishes a quantitative evaluation framework for scientific analysis. This benchmark reveals priority region where DLSR models excel, identifying specific imaging conditions under which computational inference can reliably complement or surpass conventional hardware-based SIM. At the algorithmic level, the Deep Fourier Channel Attention Network (DFCAN) introduces the Fourier Channel Attention (FCA) mechanism to resolve high-frequency biological details. Unlike traditional spatial domain approaches, FCA leverages the power spectrum of feature maps to adaptively rescale weightings based on frequency content differences. This strategy allows the model to learn precise hierarchical mappings from diffraction-limited inputs to super-resolved outputs, effectively modeling the interdependencies of informative features in the Fourier domain to minimize reconstruction artifacts even under low-SNR conditions.Implementation of these algorithms is supported by advanced hardware control utilizing a ferroelectric SLM for precise pattern generation and phase manipulation. By modulating diffraction orders and the synchronization of illumination phases, the system achieves multimodal SR imaging—including TIRF-SIM and nonlinear SIM—within a single platform. As a computational image restoration framework, DFCAN/DFGAN focuses on DL-based SIM reconstruction without hardware-based wavefront sensing or correction. The resulting DFCAN/DFGAN framework demonstrates superior robustness in live-cell imaging, extending observation durations tenfold while resolving the dynamic interactions of mitochondrial cristae and nucleoids that are typically obscured by phototoxicity or noise in conventional SR modalities (Fig. 3J) (Table 1) [58].

**Table 1:** Summary of AO-SIM Frameworks

Framework	Hardware and optical AO Strategy	Computational and deep-learning strategy	Performance and capabilities	Reference
TACO (Tandem Aberration Correction Optics)	Dual-stage AO: DM in detection path (pupil AO) and SLM in illumination path (conjugate AO). Sensorless, image-based sensing.	SPGD algorithm for iterative optimization; Gaussian kernel updates to smooth phase topography and accelerate convergence.	Resolves actin branches at 155 nm; eliminates artifacts and restores optical sectioning in complex biological media.	[62]
CNN-based Sensorless AO	SLM for wavefront correction; replaces hardware wavefront sensors.	AlexNet-based CNN maps distorted patterns to Zernike modes (4th–15th); achieves sensing in 10.1 ms.	Correlation index of 0.9986; 2.57x improvement in post-correction correlation; enhances subcellular structural definition.	[63]
MOSAIC	Dual-pathway AO (SLMs and DMs) for independent excitation/detection compensation; direct wavefront sensing.	CARE for denoising; tiled reconstruction for large FOV; PetaKit5D (GPU-accelerated) for real-time 4D processing.	Achieves resolution for mitochondrial segmentation in zebrafish embryos; facilitates quantitative 4D observations.	[64]
MLS-SIM (Multiplexed Line-Scanning)	Multiplexed/patterned line excitations; high-speed architecture (1 ms acquisition).	Row-by-row image registration; 2D cross-correlation and Gaussian kernel fitting; joint deconvolution of raw image strips.	Lateral resolution of 150 nm (linear) to 100 nm (nonlinear); immune to global sample motion (> 100 μm/s).	[65]
Hardware-based Four-beam Interference	Four-beam interference; bead-based sensing; closed-loop feedback via piezoelectric mirror/stage; RI-matched immersion.	Multi-step DL: Six-direction isotropization model; dual-stage 3D RCAN for high-fidelity denoising.	160 nm axial (hardware) and 120 nm isotropic (DL) resolution; 2–3 fold enhancement over state-of-the-art LLS.	[66]
IDDR-SPIM	Static wavefront engineering via double-ring modulation plate; Bessel-type light sheet with suppressed side lobes.	Divide-and-conquer strategy (denoising, deblurring, depixelization); DRUNet; self-supervised axial-to-lateral learning.	Approximately 100 nm isotropic resolution; 17 Hz volumetric rate; minimal photobleaching (< 0.01% per volume).	[67]
rDL (rationalized DL)	Compatible with AO; robust against wavefront errors up to 0.4λ.	Three-branch design (PFE, MPE, FCD); integrates physical priors (PSF/SI patterns); TiS-rDL/SiS-rDL self-supervised denoising.	Supports imaging at 684 fps; highly resilient to optical aberrations (resolution degradation < 2.3%).	[60]
DPA-TISR	Digital AO layer for global/local phase shift compensation.	Recurrent Network-based Propagation (RNP); Deformable Phase-space Alignment (DPA) using RFFT; Bayesian uncertainty quantification.	Validated over 10,000 timepoints; captures rapid dynamics like mitochondrial cristae transformations with high fidelity.	[68]
VSI-SR (Virtual SIM)	Reflective light-sheet configuration for dual symmetrical views.	DNN for 1D SR intermediates; MAML meta-learning for fast adaptation; RL-DFN (Richardson-Lucy dual-cycle fusion).	120 nm lateral / 160 nm axial resolution; 720-fold reduction in training time; high-speed 4D organelle imaging.	[69]
DFCAN/DFGAN	Ferroelectric SLM for high-speed pattern generation and phase manipulation.	Fourier Channel Attention mechanism; models interdependencies of features in the frequency domain.	10x extended observation duration; resolves mitochondrial cristae and nucleoids under low-SNR conditions.	[58]

## AO-SMLM: achieving nanoscale precision in deep tissue

SMLM achieves the super-resolution imaging by computationally localizing thousands of individual fluorescent molecules. This technique, however, is sensitive to optical aberrations, which distort and broaden the single-molecule PSF. In deep tissue, these distortions not only degrade localization precision but also introduce systematic errors, leading to structural artifacts in the reconstructed image. The integration of AO counteracts this, but the stochastic and sparse nature of SMLM data has required the development of correction strategies. The combination of AO with advanced PSF modeling and DL now represents a useful approach for overcoming the limitations of 3D super-resolution imaging in deep tissues.

The integration of AO into the SMLM pipeline has evolved from simple hardware correction to a sophisticated information-based framework where the PSF is utilized as an intrinsic wavefront sensor. Advanced strategies now employ In-situ PSF Retrieval (INSPR) and Phase Retrieval algorithms to extract realistic 3D-PSF models directly from raw blinking fluorophore data, bypassing the systematic errors introduced by static, bead-based calibration. By parameterizing the complex-valued pupil function through Zernike polynomials, these methods can characterize depth-dependent aberrations with high fidelity, ensuring that 3D localization precision consistently approaches the theoretical Cramer-Rao lower bound (CRLB) even in heterogeneous specimens. Furthermore, the synergy between PSF engineering and computational AO allows for the deliberate modification of the PSF shape—such as the double-helix or astigmatic PSFs—to encode precise axial information across extended volumetric ranges. In this integrated workflow, the AO system does not merely flatten the wavefront but adaptively optimizes the PSF model used for maximum likelihood estimation fitting. This aberration-aware localization approach effectively corrects for depth-induced position biases and axial scaling errors, enabling the faithful 3D reconstruction of complex structures, such as the spectrin scaffold in axons or synaptonemal complexes in *C. elegans*, which would otherwise be distorted by refractive index mismatches.

**Fig. 4:**
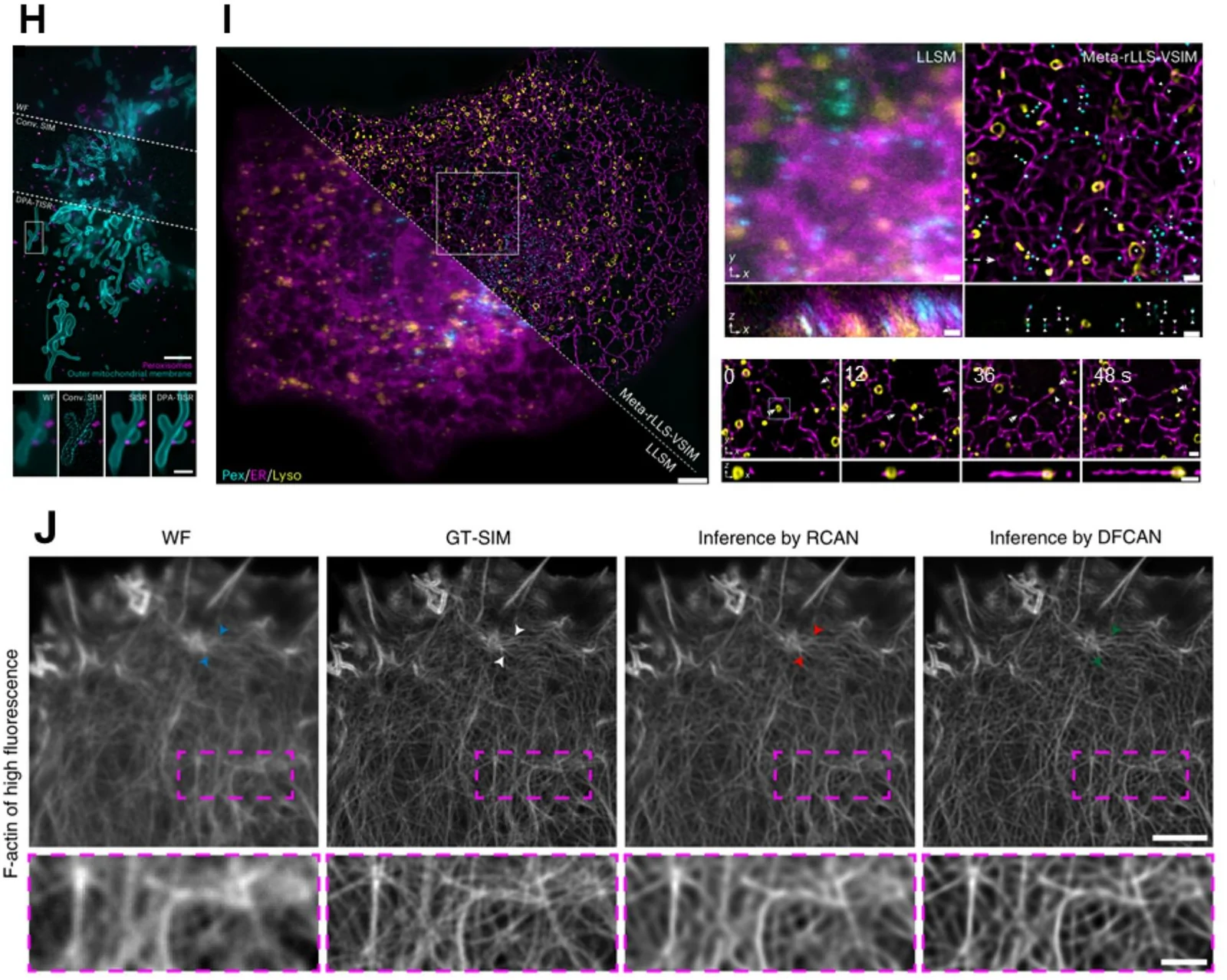
AO-SMLM. **A** SMLM with DL-AO applied in 125 μm thick 5XFAD mouse brain sections reconstructs three-dimensional structure of amyloid-β fibrils. Adapted from Zhang P, Ma D, Cheng X, Tsai AP, Tang Y, Gao H-C, et al. Nat Methods. 2023;20(11):1748–58 [70]. **B** Direct linkage between functional analysis and super-resolution structural imaging by visualizing the nanostructure of βIV-spectrin using REALM-based 3D SMLM. Scale bar: 20 mV, 500 pA, 10 ms (upper second panel, from top to bottom), 20 mV, 200 pA, 200 ms (upper fourth panel, from top to bottom), 100 μm (lower first panel), 20 μm (lower second panel), 2 μm (lower third panel). Adapted from Siemons ME, Hanemaaijer NAK, Kole MHP, Kapitein LC. Nat Commun. 2021;12(1):3407 [71]. (**C**) Combined use of AO correction and SOLEIL illumination in single-neuron SMLM of adult Drosophila brain enhances axial and lateral resolution as well as fine structure reproducibility. Adapted from Hung S-T, Llobet Rosell A, Jurriens D, Siemons M, Soloviev O, Kapitein LC, et al. Front Neurosci. 2022;16:954949 [72]. **D** Using CLASS-based AO-SMLM to acquire super-resolution imaging of cilia, oligodendrocytes, and early and mature myelination structures in intact zebrafish larvae. Scale bar: 500 μm (panel b, left), 50 μm (panel b, right), 500 nm **c**, 5 μm (**d**), and 500 nm (**e**), 500 nm (**f**), 2.5 μm (**g**), 500 nm (**h**), 500 nm (**i**), 2.5 μm (**j**), 500 nm (**k**), 500 nm (**k**), 5 μm (**m**). Adapted from Park S, Jo Y, Kang M, Hong JH, Ko S, Kim S, et al. Nat Commun. 2023;14(1):4185 [73]. **E** Implementing fine structures with improved contrast and resolution regardless of depth by processing 20 Z-plane images of COS-7 cell tubulin through AO-based remote focus control with fourth-order SOFI. Adapted from Navikas V, Descloux AC, Grussmayer KS, Marion S, Radenovic A. Nanophotonics. 2021;10(9):2451–8 [74]. **F** Applying an in situ PSF model in three-color SMLM imaging of *C. elegans* synaptonemal complex minimizes z-dependent distance measurement deviations. Scale bar: 10 μm (upper panel), 1 μm (inset in upper panel), 5 μm (lower left panel), 1 μm (lower right panel). Adapted from Liu S, Chen J, Hellgoth J, Müller L-R, Ferdman B, Karras C, et al. Nat Methods. 2024;21(6):1082–93 [75]

The DL-AO framework addresses the fundamental limitations of specimen-induced aberrations in SMLM by bypassing traditional, iterative metric-based optimization. At the core of this methodology is a deep neural network (DNN), referred to as smNet, which monitors the stochastic emission patterns of individual molecules to directly infer shared wavefront distortions. The architecture utilizes convolutional layers and residual blocks with PReLU activations and batch normalization to map individual PSFs to a vector of 28 native mirror deformation modes. By utilizing the PSF itself as an intrinsic source for aberration measurement, the system remains invariant across diverse sample structures and avoids the background artifacts associated with external guide stars. To ensure robust hardware control, the network’s estimates are processed through a Kalman filter, which recursively combines wavefront measurements before and after each correction to reduce estimation uncertainty. This stabilized feedback loop drives a DM to compensate for complex wavefront distortions, including those induced by refractive index mismatch, in near real-time with an average forward propagation speed of 0.1 s. Unlike conventional indirect AO methods that require hundreds of cycles, DL-AO achieves full compensation within 3–20 mirror updates. This high temporal resolution and high-speed inference enable the system to respond autonomously to sudden or dynamic aberration changes during continuous SMLM acquisition. The practical efficacy of this system is demonstrated in its ability to resolve the 3D ultrastructure of amyloid-β fibrils within 125 μm-thick brain sections of 5XFAD Alzheimer’s disease mouse models (Fig. 4A). While imaging without AO results in blurred clusters and distorted axial distributions due to resolution loss at depth, DL-AO restores the PSFs toward the instrument optimum. This restoration allows for the clear visualization of individual amyloid fibrils entangling to form plaques, yielding precise measurements of fibril widths at approximately 52$\:\pm\:$9 nm laterally and 72$\:\pm\:$19 nm axially. DL-AO serves as a computational tool in deep, high-fidelity tissue microscopy, supporting the structural analysis of complex neural tissues [70].

Deep-tissue SMLM is limited by sample-induced aberrations that distort the PSF and compromise localization precision. The indirect AO methods often fail in this context due to the stochastic nature of fluorophore blinking and low signal-to-background ratios (SBR). REALM overcomes these challenges by employing an indirect wavefront sensing metric, termed M_3_, which prioritizes low spatial frequencies (< 1NA/λ) where the SNR is higher than in the noise-dominated high-frequency regime. This Fourier-based approach ensures robust metric evaluation even under extreme conditions with an SBR as low as 0.0003. The correction process utilizes a DM and a model-based modal optimization algorithm to systematically compensate for Zernike-mode distortions. Unlike noise-sensitive heuristic algorithms, REALM applies a sequence of biases to individual Zernike modes and fits the resulting M_3_ values to a Gaussian function with an offset to analytically determine the optimal mirror state. By performing two to three iterative correction rounds (approximately 300 acquisitions), the algorithm effectively manages the inter-modal dependencies of aberrations, restoring PSF symmetry and enabling accurate 3D localization deep within complex tissues. Computationally, REALM is optimized for speed and stability through the use of the Fast Hartley Transform for rapid Fourier metric calculations and the implementation of fitting constraints to prevent outliers from inducing erroneous corrections. The biological utility of this framework is demonstrated by resolving the periodic βIV-spectrin scaffold at the axon initial segment at depths up to 50 μm in brain sections. This capability facilitates a direct correlation between the functional data obtained via electrophysiological patch-clamp recordings and the underlying nanoscale architecture of functionally identified neurons (Fig. 4B) [71].

The AO-SOLEIL framework integrates a single-objective lens inclined light-sheet (SOLEIL) architecture with indirect AO to facilitate high-fidelity 3D SMLM in thick biological specimens. By utilizing an oblique light-sheet profile synchronized with a virtual confocal rolling shutter readout, SOLEIL effectively suppresses out-of-focus background fluorescence, which is a primary deterrent to localization precision in scattering environments like the adult Drosophila brain. This optical sectioning capability, achieved without the need for specialized sample holders, provides the high-contrast raw data necessary for accurate sub-cellular reconstruction at depths where standard widefield illumination typically fails due to poor SBR. Wavefront control is governed by a model-based indirect AO strategy that optimizes the seven Zernike modes ($\:{Z}_{2}^{\pm\:2}$, $\:{Z}_{3}^{\pm\:1}$,$\:\:{Z}_{3}^{\pm\:3}$,$\:\:{Z}_{4}^{0}$) by maximizing a Fourier annulus image quality metric. The correction process utilizes 11 measurement steps per mode, where the optimal state is derived from a Gaussian function fit of the metric values, and a deterministic calibration compensates for the inherent cross-talk between defocus and spherical terms. This active hardware correction is synergistically paired with In-situ PSF Retrieval (INSPR) and maximum likelihood estimation (MLE) to refine the 3D-PSF model directly from raw blinking data. Collectively, these computational and optical methodologies yield a two-fold improvement in axial localization precision and eliminate depth-induced position biases, enabling the visualization of fine structural details in deep tissue in Fig. 4C [72].

To overcome the inherent depth limitations of SMLM caused by specimen-induced aberrations, this study introduces a label-free AO framework utilizing closed-loop accumulation of single-scattering (CLASS) microscopy. Unlike conventional indirect AO, which relies on iterative optimization of fluorescence metrics and is prone to photobleaching, The CLASS identifies complex wavefront distortions by measuring the intrinsic reflectance of tissue structures. This approach bypasses the need for artificial guide stars or fluorescent particles, enabling high-fidelity imaging in environments where single-molecule PSFs are otherwise completely obscured by severe aberrations. The system’s performance is rooted in a robust computational strategy centered on reflection matrix analysis and linear optimization. By recording multiple interferometric reflectance images at diverse illumination angles, the CLASS algorithm reconstructs a reflection matrix and applies a linear optimization process to find a phase mask that maximizes single-scattering intensity. This identifies aberrations with a correction speed faster than previous modalities. The calculated correction map is then displayed on a SLM in the emission path to physically compensate for root-mean-square wavefront distortions of up to 3.08 rad - roughly three times the limit of current state-of-the-art AO-SMLM. This integration of label-free sensing and physical correction enables the restoration of near-ideal PSFs at depths of up to 102 μm within whole zebrafish larvae. The computational restoration leads to a localization number enhancement of up to 37.4-fold and improves localization precision by 2.32 to 3.61 times, achieving a quality comparable to aberration-free samples. By resolving sub-diffraction structures such as the 180 nm spacing of ciliary membranes and 120 ~ 140 nm nascent myelin sheaths, this AO-SMLM platform demonstrates its potential for advanced investigation in developmental biology and neurobiology at the whole-organism level in Fig. 4D [73].

The implementation of an AO architecture within a widefield microscope system enables a multimode approach to 3D super-resolution imaging. By utilizing a DM for synchronized remote focusing and aberration correction, an axial range of 10 μm is achieved while keeping the specimen stationary. This configuration, supported by an active infrared Z-stabilization feedback loop, eliminates the mechanical instabilities and axial drift associated with physical objective or stage movement. Such a design allows for high-speed volumetric acquisition—with plane switching frequencies up to 100 Hz—facilitating the homogeneous distribution of photobleaching throughout the sample volume. The framework’s wavefront control strategy utilizes a hybrid sensing and correction routine to mitigate both system-induced and sample-induced distortions. Initial system calibration is performed with a Shack-Hartmann wavefront sensor to establish a precise interaction matrix. For in-situ specimen imaging, a sensorless algorithm iteratively optimizes the first 15 Zernike modes by maximizing an image-based contrast metric. To maintain PSF integrity across the full axial range, third-order spherical aberrations are computationally compensated (60 nm RMS 10 μm), while axial scanning is driven by the dynamic modulation of the Zernike defocus mode. Volumetric reconstruction is facilitated by advanced computational algorithms, including spatiotemporal correlation for super-resolution optical fluctuation imaging (SOFI) and weighted least-squares Gaussian PSF fitting for 3D SMLM. As demonstrated in Fig. 4E, this algorithmic synergy ensures uniform lateral resolution (approximately 75 nm) even at an imaging depth of 9.9 μm. These results confirm that AO-based correction effectively bridges the resolution gap between the coverslip interface and deep cellular structures [74] (Table 2).

The universal inverse modeling of PSF (uiPSF) framework represents a modular toolbox for inferring accurate PSF models directly from microscopy data, including fluorescent bead stacks and in situ blinking fluorophores. It leverages Fourier-domain parametrization where the complex-valued pupil function is decomposed into an orthogonal basis of Zernike polynomials to achieve robust wavefront sensing. This approach enables the precise characterization of system- and sample-specific aberrations, which are subsequently compensated through hardware-based direct AO using DMs or through software-based refinement during the 3D localization process. At its computational core, uiPSF employs an iterative inverse modeling strategy that minimizes the discrepancy between a parameterized forward model and noisy, pixelated experimental observations. Implemented within the TensorFlow environment, the framework utilizes automatic differentiation to optimize a vast parameter space—including voxel values, Zernike coefficients, and emitter positions—without requiring manual derivative calculations. The optimization typically employs the L-BFGS algorithm and a maximum-likelihood-based loss function to account for Poisson shot noise, ensuring that 3D localization precision consistently approaches the theoretical CRLB. The versatility of uiPSF extends to spatially variant PSF modeling, where field-dependent (FD) aberration maps are reconstructed to facilitate high-throughput, whole-cell 3D imaging over large fields of view. The framework exhibits significant synergy with DL algorithms, such as the FD-DeepLoc fitter, by providing the rigorous physical models necessary for training and validating neural networks in complex biological environments. Furthermore, compared to existing phase-retrieval methods like INSPR, the uiPSF approach demonstrates superior accuracy and structural fidelity, particularly in reducing axial localization biases and handling deep-tissue imaging artifacts. This was demonstrated through three-color SMLM imaging of synaptonemal complex (SC) proteins in the *Caenorhabditis elegans* (*C. elegans*) gonad. By comparing distance measurements derived from a bead-based PSF calibration versus an in situ model, researchers showed that the in situ approach minimized depth-dependent deviations. This enabled precise and reliable measurements of the nanoscale organization of SC components, proving that such computational correction strategies are essential for extracting accurate data from deep-tissue SMLM (Fig. 4F) [75].

**Table 2:** Summary of AO-SMLM Frameworks

Framework	Hardware and Optical AO Strategy	Computational and Deep-Learning Strategy	Performance and Capabilities	Reference
DL-AO (smNet)	DM controlled via a stabilized feedback loop with a Kalman filter to reduce estimation uncertainty.	smNet (Deep Neural Network) utilizing CNNs and residual blocks to map individual PSFs to 28 mirror deformation modes; inference speed of 0.1 s.	Resolves 3D ultrastructure of amyloid-β fibrils in 125 μm-thick brain sections; achieves full compensation within 3–20 mirror updates.	[70]
REALM	DM-based model-based modal optimization using a sequence of Zernike mode biases.	M_3_ Fourier-based metric (low-frequency priority) and Fast Hartley Transform; analytically determines optimal mirror states via Gaussian fitting.	Robust performance at extremely low SBR (0.0003); resolves βIV-spectrin scaffolds at 50 μm depth in brain tissue.	[71]
AO-SOLEIL	Single-objective inclined light-sheet (SOLEIL) with virtual confocal rolling shutter and indirect AO (7 Zernike modes).	Fourier annulus image quality metric combined with In-situ PSF Retrieval (INSPR) and MLE for refined 3D-PSF modeling.	Two-fold improvement in axial localization precision; effectively suppresses background in scattering environments like the Drosophila brain.	[72]
CLASS	Label-free sensing via intrinsic tissue reflectance; uses a SLM in the emission path for physical correction.	Reflection matrix analysis and linear optimization to identify phase masks that maximize single-scattering intensity.	Corrects RMS distortions up to 3.08 rad; enables imaging at 102 μm depth in zebrafish larvae with up to 37.4-fold localization enhancement.	[73]
Multimode Widefield AO	DM for synchronized remote focusing (10 μm range) and active infrared Z-stabilization; includes Shack-Hartmann sensor for initial calibration.	Sensorless iterative optimization of 15 Zernike modes; synergistic use of SOFI and weighted least-squares Gaussian PSF fitting.	High-speed volumetric acquisition (100 Hz); maintains uniform lateral resolution (approximately 75 nm) at depths up to 9.9 μm.	[74]
uiPSF	Hardware-based direct AO (DM) or software-based refinement; leverages complex-valued pupil function parametrization.	TensorFlow-based iterative inverse modeling using automatic differentiation and L-BFGS optimization; accounts for field-dependent (FD) aberrations.	Approaches theoretical CRLB; enables accurate three-color SMLM of synaptonemal complexes in C. elegans with minimal axial bias.	[75]

## AO-STED microscopy: preserving nanoscale resolution in scattering tissue

STED microscopy achieves super-resolution by using a donut-shaped depletion beam to confine fluorescence emission to a sub-diffraction-sized region. The efficacy of this technique hinges critically on the quality of the zero-intensity minimum at the center of this donut. In deep tissues, however, sample-induced aberrations are pernicious to STED. They can distort the depletion beam, or more severely, fill in the central minimum with light. This effect eliminates fluorescence confinement, causing a complete loss of both resolution and signal, effectively reverting the microscope’s performance to that of a low-efficiency confocal system. The integration of AO provides a direct and potent solution to this vulnerability. The AO-STED preserves the integrity of the depletion donut deep within scattering samples, ensuring a tight, dark central minimum and thereby restoring high resolution and signal levels. This capability has been foundational in moving STED from an application restricted to cultured cells to a useful tool for in vivo biology.

In STED microscopy, the operational role of AO is prioritized within the depletion beam path due to the extreme sensitivity of the donut-shaped focus to phase distortions. Aberrations in the depletion path can lead to a non-zero central intensity, which catastrophically reduces resolution by prematurely depleting the central fluorescence. To address this, AO modules are often implemented as online hardware loops that provide real-time compensation for the depletion wavefront while simultaneously ensuring the spatial overlap of the excitation and depletion foci. While some architectures employ a single DM to correct all beams concurrently, more complex systems utilize a secondary SLM for finer, high-order correction of the critical depletion beam, thereby maintaining the physical integrity of the central null deep within scattering tissues.

The efficacy of resolution enhancement in STED microscopy is fundamentally contingent upon the physical integrity of the depletion beam’s zero-intensity central null. Sample-induced aberrations are particularly pernicious in this modality, as even minor phase distortions can cause fill-in of the central minimum, leading to premature fluorescence depletion and a catastrophic loss of both spatial resolution and SBR. Within a unified AO framework, correction is strategically distributed across the excitation, depletion, and detection paths. While a single DM positioned in a common pupil plane can provide simultaneous first-order correction for all co-aligned beams, advanced architectures frequently utilize a secondary SLM dedicated to the depletion beam. This dual-modality approach allows for independent, high-order modulation of the depletion phase mask, ensuring that the hollow PSF remains undistorted even deep within scattering media where complex refractive index mismatches occur.

The implementation of AO in the STED workflow spans from deterministic, a priori calibration to dynamic, online feedback loops. Deterministic strategies utilize depth-dependent models to apply instantaneous spherical aberration corrections via an SLM, effectively bypassing time-consuming iterations and minimizing phototoxicity during volumetric acquisition. For more complex and dynamic specimen-induced distortions, online sensorless AO routines iteratively optimize image-quality metrics—such as brightness and sharpness—by refining Zernike polynomial coefficients on the wavefront correction device. This synergy with DL computational frameworks contributes to automation by allowing accelerated aberration estimation from single frames, thereby reducing the multiple hardware loops. The hybrid hardware-software frameworks, such as dynamic phase switching, enable real-time background recording and subtraction by modulating the depletion pupil function, preserving nanoscale fidelity across large 3D volumes in living systems.

**Fig. 5:**
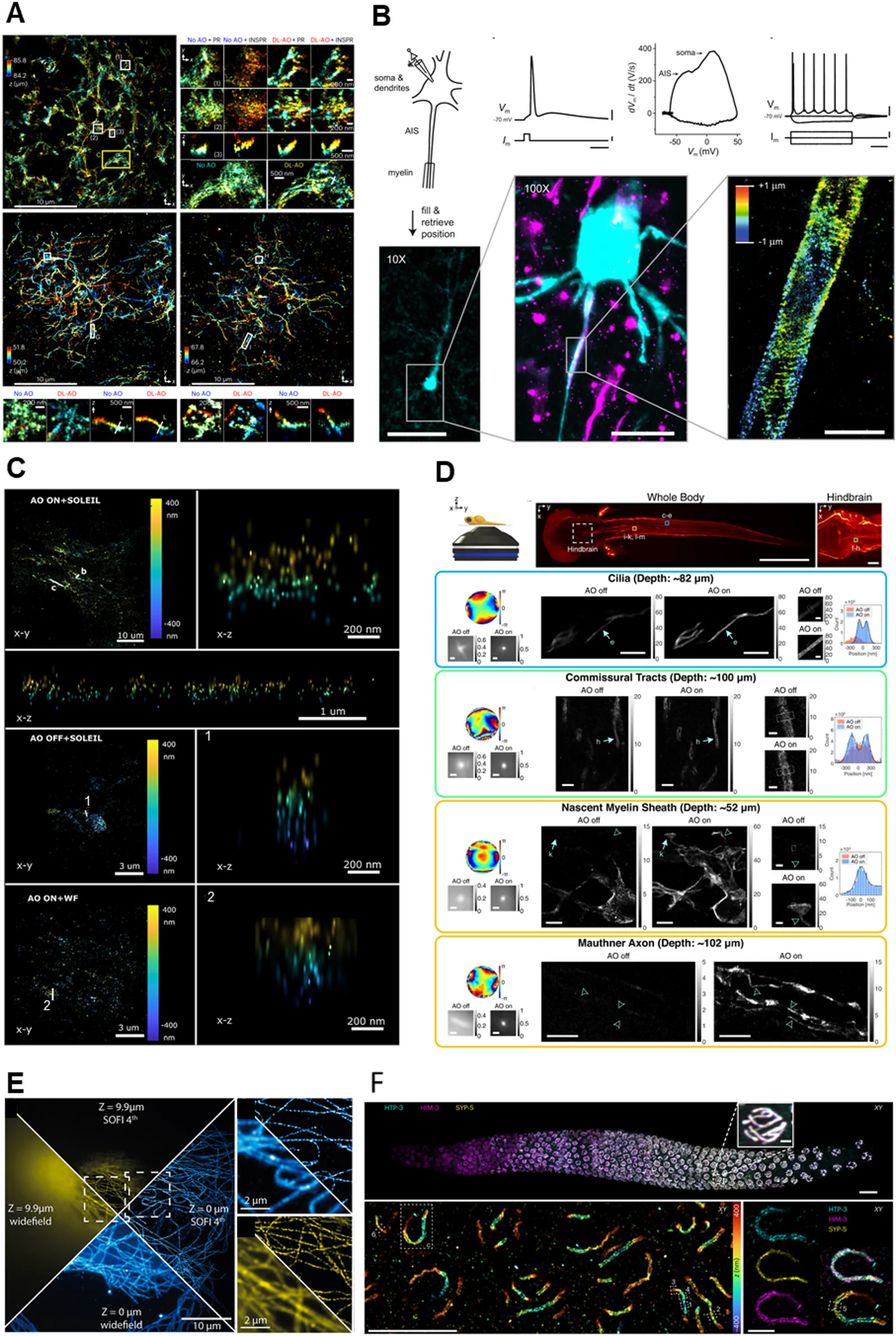
AO-STED. **A** AO 3D-STEDD improves the resolution as well as background removal performance compared to confocal and STEDD without AO application. Scale bar: 2 μm. Adapted from Dai S, Kobitski A, Barati Sedeh A, Eroğlu-Kayıkçı S, Hilbert L, Nienhaus GU. ACS Photonics. 2024;11(1):310–20 [76]. **B** isoSTED microscopy with AO correction can visualize fine structures of organelles and synaptic proteins. Scale bar: 1 μm (left panel), 100 nm (right panel). Adapted from Hao X, Allgeyer ES, Lee D-R, Antonello J, Watters K, Gerdes JA, et al. Nat Methods. 2021;18(6):688–93 [77]. **C** Prior spherical aberration correction mitigates PSF deterioration and signal reduction in deep STED imaging of acute hippocampal sections. Adapted from Stéphane B, Luc M, Emanuele M, Nägerl UV. Neurophotonics. 2021;8(3):035001 [78]. **D** AO-based wavefront correction enhances axial resolution and fine chromatin structure restoration in deep mouse skin tissue. Scale bar: 1 μm. Adapted from Velasco MGM, Zhang M, Antonello J, Yuan P, Allgeyer ES, May D, et al. Optica. 2021;8(4):442–50 [79]. **E** 3D STED microscopy with dual SLM-based AO applied to human induced pluripotent stem cell (hiPSC)-derived neuronal axons grown on 80 μm high-aberration tissue achieves lateral and axial resolutions of 182 nm and 310 nm, respectively. Scale bar: 2 μm (unless stated otherwise). Adapted from Zdańkowski P, Trusiak M, McGloin D, Swedlow JR. ACS Nano. 2020;14(1):394–405 [80]. **F** DPS-STED suppresses background signal in U2OS cell microtubule imaging, achieving more than a threefold improvement in SBR compared to conventional 3D-STED. Adapted from Tu S, Liu X, Yuan D, Tao W, Han Y, Shi Y, et al. ACS Photonics. 2022;9(12):3863–8 [81]. **G** Self-Net achieves three-dimensional isotropic super-resolution by enhancing axial resolution to lateral resolution levels in STED and SIM imaging. Scale bar: 1 μm (upper panel), 0.5 μm (lower three panels). Adapted from Ning K, Lu B, Wang X, Zhang X, Nie S, Jiang T, et al. Light Sci Appl. 2023;12(1):204 [82]. **H** RCAN enhances confocal images to STED-level quality, achieving approximately two-fold resolution improvement and noise reduction in microtubules, nuclear pore complexes, and DNA structures. Scale bar: 5 μm. Adapted from Chen J, Sasaki H, Lai H, Su Y, Liu J, Wu Y, et al. Nat Methods. 2021;18(6):678–87 [45]

Deep-tissue 3D-STED imaging at depths exceeding 100 μm is limited by sample-induced aberrations that degrade the focal intensity distribution of depletion beams, particularly the sensitive bottle beam. To mitigate this, a photon-efficient, indirect modal AO strategy was developed using the reflection imaging of 80 nm gold particles as fiducial markers. By quantifying wavefront sensing through the scattered light of these beads at specimen boundaries, the system avoids exposing the sample to high-intensity depletion beams, thereby minimizing phototoxicity and photobleaching during the optimization process. The wavefront correction algorithm operates by decomposing phase distortions into orthogonal Zernike polynomials, which are then compensated for using dual SLMs in the 2D-STED and z-STED paths. An aspect of this computational routine is the management of mode coupling in high-NA systems, where primary aberrations like coma and spherical modes are intrinsically linked to displacement modes such as tip, tilt, and defocus. To prevent the misalignment of excitation and depletion foci, the algorithm applies coupled Zernike modes at specific amplitude ratios designed to minimize focal shifts while maximizing the cross-correlation between experimental and ideal PSFs. A fully automated computational framework facilitates rapid parameter optimization through parabolic fitting of the cross-correlation metrics for each Zernike mode. The strategy utilizes a linear interpolation model for correction parameters between zero and maximum sample depths, enabling reliable wavefront compensation throughout the heterogeneous tissue volume without requiring repetitive exposures. When integrated with background-free stimulated emission double depletion (STEDD) microscopy, this approach maintains a three-fold axial resolution enhancement at depths of 110 μm, as demonstrated by the high-fidelity imaging of RNA Polymerase II clusters in zebrafish embryos in Fig. 5A [76].

The AO isoSTED microscopy integrates a vertically oriented 4Pi interference cavity with dual-objective STED microscopy to achieve sub-50-nm isotropic resolution in thick biological specimens. Central to this system is a sensorless AO architecture that overcomes depth-dependent aberrations by treating the counter-propagating beam paths as a unified optical system via a specialized 4Pi aberration model. The wavefront sensing is performed indirectly through an image-based metric optimization, where the system iteratively probes Zernike modes to maximize fluorescence intensity or PSF quality without the need for a physical wavefront sensor. The wavefront correction is executed by two DMs positioned in conjugate pupil planes, controlled through a computationally derived influence matrix that maps actuator voltages to desired Zernike coefficients. A multistep compensation protocol is employed to sequentially eliminate system-induced misalignments, coarse sample-induced aberrations from refractive index mismatches, and fine-scale 4Pi phase distortions. This computational approach utilizes a least-squares optimization to determine the precise DM surface shapes required to counteract complex wavefront distortions, effectively restoring a nearly ideal, side-lobe-free effective PSF deep within tissue. To maintain axial stability during 3D volumetric scanning, the system incorporates a linear regression-based bias compensation algorithm that adjusts for depth-dependent shifts in interference fringes. Furthermore, image decorrelation analysis is used to rigorously quantify the achieved resolution, ensuring the reliability of structural data such as the 100 nm spacing between Bassoon and Homer1 markers in 30 μm-thick mouse hippocampal sections. This synergistic integration of AO control and deterministic optimization algorithms enables high-fidelity nanoscopic imaging of subcellular organizations previously obscured by scattering and aberrations in thick specimens in Fig. 5B [77].

The challenge in deep-tissue STED microscopy is the catastrophic degradation of the depletion beam’s central null due to sample-induced spherical aberrations. To bypass the limitations of typical AO, this study utilizes an a priori calibration strategy using an agarose phantom gel with a refractive index of 1.38, closely mimicking living brain tissue. By quantifying the necessary correction as a function of depth before imaging the biological specimen, the authors established a robust framework that achieves nanoscale resolution (80 nm) at a depth of 90 μm within living brain slices. This method effectively circumvents the need for complex, time-consuming real-time measurements that often lead to sample degradation in deep-tissue settings. The wavefront sensing protocol in this work employs a sensorless, image-based readout that leverages the unique sensitivity of STED PSFs to optical distortions. Instead of a hardware-based sensor, the system monitors the morphological deformations of donut and bottle beams reflected from gold nanospheres. These distortions are mathematically decomposed using Zernike polynomials, identifying spherical aberration as the dominant factor. Correction is executed via a SLM, which applies a pre-distorted phase mask to the depletion beam, neutralizing aberrations at the focal plane to restore the high-contrast central null required for super-resolution. A critical advantage of this methodology is its reliance on a deterministic linear computational model rather than iterative, black-box optimization algorithms like genetic algorithms. By mapping the spherical correction coefficient to depth, the system provides instantaneous correction without the photobleaching or phototoxicity associated with iterative closed-loop cycles. This computational efficiency enabled the successful morphometric analysis of dendritic spine necks in the hippocampus, yielding an average width of 160$\:\pm\:$15 nm - a measurement previously unattainable at such depths without AO intervention. The approach was validated using the SpineJ plugin, demonstrating that mimicking the brain as a bulk medium is sufficient for significant resolution gains (Fig. 5C) [78].

Recent advancements in deep-tissue microscopy have integrated two-photon excitation with direct wavefront sensing AO to circumvent the resolution degradation typically encountered in thick biological specimens. By utilizing near-infrared (NIR) wavelengths for both excitation and depletion, this configuration significantly reduces light scattering while employing a long-working-distance water-immersion objective to minimize refractive index mismatches between the immersion medium and the specimen. This synergistic approach enables 3D-STED imaging with a lateral resolution of 70 nm and an axial resolution of 151 nm, facilitating sub-diffraction visualization at depths up to 164 μm in fixed tissue and 76 μm within the intact living mouse brain. The adaptive optics architecture relies on a nonlinear guide star strategy, where 2PE-induced fluorescence serves as a localized point source to probe specimen-induced phase distortions. A SH-WFS captures the aberrated wavefront, which is subsequently processed by a modal decomposition algorithm to reconstruct the phase error into its constituent Zernike polynomials. Through a closed-loop feedback system, the calculated inverse phase profile is applied to a DM, iteratively refining the correction to restore the integrity of the top-hat and vortex depletion foci. This active compensation is essential for maintaining the zero-intensity minimum of the STED beam, which is otherwise compromised by the optical heterogeneities of deep tissue. Beyond hardware-based correction, advanced computational frameworks are employed to ensure the quantitative validity of the super-resolution data. A nested-loop ensemble PSF fitting algorithm is utilized for resolution quantification, which accurately accounts for the finite geometry of the target structures—such as microtubules or dendritic spines—to provide a more rigorous assessment than conventional Gaussian fitting. Furthermore, to manage the physiological challenges of in vivo imaging, offline image registration algorithms are implemented to mitigate jittering artifacts caused by animal respiration and cardiac cycles. These integrated computational strategies, combined with active phase control, ensure that nanometer-scale morphological changes can be tracked with high fidelity in complex, intact living systems in Fig. 5D [79].

The research demonstrates a custom-built 3D STED microscope integrating dual SLMs to achieve SR imaging at significant depths within scattering biological tissues. By implementing AO, the system resolves axons of human induced pluripotent stem cell (hiPSC)-derived neurons situated under an 80 μm thick tissue layer. The architecture allows for independent control of excitation and depletion beams, effectively overcoming sample-induced aberrations that typically degrade STED performance in thick specimens. The wavefront sensing and correction protocol utilizes a modal approach based on Zernike polynomials to compensate for both optical system and specimen-induced aberrations. Specifically, the system corrects first-order astigmatism, coma, trefoil, and up to third-order spherical aberrations. For the excitation beam, the algorithm maximizes a quality metric—defined as the square of the total image intensity—to optimize focal quality. Conversely, depletion beam correction involves a two-step process: initial alignment using gold beads followed by a minimum search of the quality metric while the beam maintains a Gaussian profile to maximize depletion efficiency. This precise modal correction facilitates a 2.5-fold improvement in axial resolution (310 nm) compared to aberration-corrected confocal modes (795 nm). To further enhance image quality, the researchers employed the block-matching and 3D filtering (BM3D) algorithm for numerical denoising, offering a robust alternative to data-intensive DL methods. BM3D exploits non-local similarities within the image to perform collaborative filtering in the 3D transform domain, effectively retrieving high-frequency structural details previously obscured by noise. This computational integration yields a 31% increase in effective resolution. The improvement is quantitatively validated by the Fourier Ring Correlation metric, demonstrating that numerical enhancement significantly extends the operational limits of deep-tissue microscopy (Fig. 5E) [80].

To mitigate the SBR degradation caused by optical aberrations and out-of-focus background in deep-tissue 3D-STED microscopy, a dual-element AO architecture is employed. This system integrates a SLM for precise depletion beam shaping and a DM in the detection path for active wavefront correction. The wavefront sensing follows a sensorless, multi-step optimization routine that iteratively adjusts the surface profile of the DM to maximize fluorescence intensity, effectively compensating for both system-intrinsic and depth-dependent specimen aberrations. The dynamic phase switching (DPS) algorithm achieves deterministic background recording by modulating the pupil function of the depletion beam. In the signal-on mode, standard vortex and top-hat phase masks generate the hollow PSF required for super-resolution imaging. Conversely, the signal-off mode switches the handedness of the vortex phase mask and modifies the inner radius of the top-hat mask to break the central zero-intensity spot. This configuration intentionally depletes the focal fluorescence, allowing the system to isolate and record a dedicated background frame that accurately represents out-of-focus incomplete depletion. Computational refinement of the final image is performed through a statistical subtraction framework designed to account for intensity fluctuations and photobleaching effects. An optimal subtraction factor is estimated using a least-squares approach that minimizes the variance between the raw image and the recorded background. Prior to subtraction, the background frame is pre-processed with a Gaussian filter to suppress shot noise associated with shortened pixel dwell times. This hybrid hardware-software approach facilitates a 3–7 fold improvement in SBR, significantly outperforming conventional algorithms such as the rolling ball or wavelet-based subtraction methods (Fig. 5F) [81].

The Self-Net framework addresses the inherent resolution anisotropy in volumetric fluorescence microscopy, where the axial resolution is typically two to three times inferior to the lateral resolution due to an asymmetric PSF. While physical wavefront sensing and hardware-based correction methods like adaptive optics can mitigate these effects, they often introduce prohibitive system complexity and cost. Self-Net provides a computational alternative by leveraging high-resolution lateral images within the same dataset as rational targets for axial restoration. This approach eliminates the requirement for prior knowledge of the image formation process or specialized hardware, effectively resolving data quality bottlenecks in large-scale biological imaging. The DL architecture utilizes a two-stage self-learning strategy designed to achieve high-fidelity reconstruction without the need for pre-registered training pairs. The first stage employs a modified CycleGAN framework to perform unsupervised degradation modeling, learning the transformation from HR lateral images to blurred axial slices. In the second stage, a dedicated recovery network called DeblurNet is trained via supervised learning, using the synthetic blurred images from the first stage as inputs and pixel-aligned lateral images as ground truth. This dual-stage design imposes a strong structural constraint on the restoration process, which significantly reduces the hallucination artifacts often associated with standard unsupervised generative adversarial networks. For enhanced performance in challenging imaging conditions, the framework integrates classical computational algorithms, such as Richardson-Lucy deconvolution, within the Self-Net+ variant to suppress out-of-focus background light and improve the SBR. Self-Net demonstrates substantial computational efficiency compared to 3D networks like OT-CycleGAN, exhibiting a 10-fold reduction in model parameters and 20-fold faster training times. With an inference speed of 1.2 s for a 200^3^-voxel volume, the method enables real-time, on-demand isotropic restoration. This capability facilitates the accurate and efficient reconstruction of complex neuronal morphologies within terabyte-scale whole-brain datasets (Fig. 5G) [82].

The 3D Residual Channel Attention Network (RCAN) addresses the inherent spatiotemporal trade-offs in fluorescence microscopy by providing a robust computational framework for volumetric image restoration. Unlike conventional 2D architectures, this model utilizes 3D convolutional layers to process image volumes, maintaining spatial consistency across the axial dimension. The core Residual in Residual (RIR) structure employs multiple long and short skip connections that allow the network to bypass abundant low-spatial-frequency information. This architecture prioritizes the learning of high-frequency structural details, enabling effective denoising and restoration even under conditions of severe photobleaching or low illumination intensity. Central to the network’s analytical performance is the channel attention mechanism, which adaptively rescales feature channels by modeling their interdependencies. This mechanism serves as a computational alternative to physical wavefront correction, effectively deblurring images and retrieving resolution degraded by the optical system’s PSF. By selectively emphasizing high-spatial-frequency features, the algorithm achieves superior sharpening compared to standard U-net or GAN-based architectures. Furthermore, training stability in sparse microscopy datasets is maintained through the use of large 3D patches (typically 256 × 256 × 8 μm), which ensure sufficient gradient information for back-propagation. The practical utility of RCAN is demonstrated through cross-modality resolution enhancement, transforming standard confocal volumes into super-resolution data. Quantitative evaluations show a 2.5-fold lateral resolution improvement in confocal-to-STED applications and up to a 3.6-fold axial enhancement when utilizing expansion microscopy ground truth. A two-step chaining strategy, which sequentially applies a denoising model followed by a resolution enhancement model, further optimizes visual and quantitative fidelity. This computational approach enables extended 4D imaging with negligible photobleaching, offering a persuasive alternative to hardware-intensive SR techniques (Fig. 5H) (Table 3) [45].

**Table 3:** Summary of AO-STED microscopy frameworks

Framework	Hardware & optical AO strategy	Computational & deep-learning strategy	Performance & capabilities	Reference
Deep-tissue 3D-STED	Dual SLMs for 2D and z-STED paths; 80 nm gold particles as fiducial markers for reflection imaging.	Indirect modal AO using Zernike decomposition; management of mode coupling (e.g., coma/spherical linked to tilt/defocus); automated parabolic fitting for optimization.	Maintains 3-fold axial resolution enhancement at depths exceeding 110 μm; imaged RNA Polymerase II in zebrafish embryos.	[76]
AO isoSTED Nanoscope	Vertically oriented 4Pi interference cavity with dual objectives; two DMs in conjugate pupil planes.	Sensorless AO based on image metric optimization; 4Pi aberration model; influence matrix for DM control; linear regression for axial fringe stability.	Achieves sub-50 nm isotropic resolution in 30 μm-thick tissue; resolves 100 nm spacing between synaptic markers (Bassoon/Homer1).	[77]
A Priori Calibrated STED	SLM for depletion beam shaping; agarose phantom (RI 1.38) for pre-imaging calibration.	Sensorless, image-based readout using gold nanosphere reflections; deterministic linear model mapping spherical aberration to depth (avoids iterative loops).	80 nm resolution at 90 μm depth in living brain slices; measured 160 nm dendritic spine necks in the hippocampus.	[78]
2P-STED with Direct AO	NIR wavelengths; long-working-distance water objective; Shack-Hartmann sensor (SHS) and DM.	Nonlinear guide star (two-photon excitation fluorescence); closed-loop feedback for phase correction; nested-loop ensemble PSF fitting; offline image registration for motion artifacts.	Lateral 70 nm / Axial 151 nm resolution; imaging depths up to 164 μm (fixed) and 76 μm (in vivo living mouse brain).	[79]
Custom 3D STED (hiPSC focus)	Custom-built system with dual SLMs for independent excitation and depletion beam control.	Modal Zernike correction (up to 3rd order spherical); BM3D (Block-matching and 3D filtering) algorithm for numerical denoising and resolution retrieval.	2.5-fold axial resolution improvement (310 nm) at 80 μm depth; 31% increase in effective resolution via numerical enhancement.	[80]
Dual-Element AO 3D-STED	SLM for depletion shaping and DM for detection path wavefront correction.	Dynamic Phase Switching (DPS) for background subtraction; sensorless multi-step optimization; statistical subtraction framework using least-squares.	Provides 3–7 fold improvement in SBR by effectively isolating out-of-focus background.	[81]
Self-Net (DL)	Computational alternative to physical AO hardware (reduces system complexity/cost).	Two-stage self-learning: CycleGAN for unsupervised degradation modeling and DeblurNet for supervised recovery; utilizes lateral images as targets.	1.2 s inference speed for large volumes; 10x reduction in parameters; enables isotropic restoration of terabyte-scale whole-brain data.	[82]
3D RCAN	3D Convolutional Neural Network (standard hardware compatible).	Residual in Residual (RIR) structure with Channel Attention mechanism; prioritizes high-frequency details; two-step denoising-enhancement chaining.	2.5x lateral and 3.6x axial enhancement (Confocal-to-STED/ExM); enables long-term 4D imaging with minimal photobleaching.	[45]

## Discussion

The integration of AO with SRM enhances deep-tissue biological imaging by addressing sample-induced aberrations and light scattering that restrict nanoscale visualization to superficial layers. By restoring sub-diffraction limited performance deep within complex biological specimens, this technological convergence enables the visualization of dynamic molecular processes within their native, physiologically relevant contexts, ranging from the synaptic architecture of the living brain to cellular morphogenesis in developing organoids.

Furthermore, the request for higher resolution and faster acquisition presents a persistent trade-off. SMLM techniques like PALM and STORM offer exceptional spatial resolution but are hampered by slow temporal resolution, often requiring tens of seconds to minutes to reconstruct a single super-resolved image, rendering them too slow for many rapid dynamic processes. While techniques such as DNA-PAINT are improving speed, they still face the challenge of achieving the requisite temporal resolution without compromising localization precision [83–85]. Conversely, STED provides real-time, reconstruction-free imaging but requires higher laser powers, increasing the risk of phototoxicity. In addition, the complexity and cost of implementing AO systems, coupled with the challenge of correcting spatially variant aberrations across a large field of view, remain barriers to making this technique a tool for the broader biological research community.

Conventional sensorless AO methods frequently exacerbate these issues, as their iterative nature demands multiple exposures that increase the risk of phototoxicity and reduce experimental throughput. DL strategies offer improvements to this field by reducing the need for multiple hardware corrections through accelerated aberration prediction and high-fidelity image restoration. This transition enables the realization of systemic autonomy through self-driving frameworks where intelligent agents optimize hardware parameters in real-time, effectively bridging the gap between computational inference and physical correction. By combining the physical signal restoration of adaptive optics with the algorithmic fidelity of content-aware restoration and the autonomous decision-making of machine learning agents, researchers can achieve a triple synergy that maximizes resolution while minimizing light exposure.

The challenge for high performance continues on multiple fronts. The development of techniques like minimal photon fluxes (MINFLUX) is pushing spatial resolution toward the single-nanometer scale, nearing the precision of electron microscopy while retaining the advantages of fluorescence imaging [86–90]. The correlative light and electron microscopy (CLEM) is emerging as a useful approach to bridge this gap, providing both the molecular specificity of SRM and the ultrastructural context of EM. These advances are converging toward a future of integrated and intelligent imaging pipelines [91–96].

We envision systems where a physical AO module provides a rapid, first-order correction, and a physics-informed neural network then takes the raw data stream to produce a fully corrected, super-resolved volumetric movie in real-time. This optimization balances imaging speed and fidelity with the required light dose, facilitating sustained, 4D nanoscale imaging of sensitive biological processes.

The SRM is undergoing an evolution, moving beyond the imaging of cultured cells to the exploration of complex, multicellular systems. The AO-SRM has been the critical step in breaking the imaging depth barrier, enabling nanoscale visualization within intact tissues. Augmented by DL, this field enables to overcome the hurdles of speed, phototoxicity, and accessibility. These integrated technologies are not just providing super-resolution images. These approaches contribute useful tools to biological inquiry. By facilitating the observation of molecular dynamics in their native context, AO-SRM supports ongoing research in neuroscience, particularly in subcellular dynamics. It is also indispensable in biomedical research for deciphering the mechanisms of disease and developing next-generation therapeutics.

The application of AO-SRM to molecular and cellular neuroscience facilitates the high-resolution characterization of sub-diffraction macromolecular assemblies deep within intact neural tissues. These techniques permit the spatial mapping of trans-synaptic nanocolumns—such as the alignment of presynaptic release sites with postsynaptic receptor domains [65, 77]—and the longitudinal tracking of structural plasticity in dendritic spine heads and necks [65, 78, 79]. Furthermore, the capability of AO-SRM to bridge the gap between structure and function is exemplified by correlating the nanoscale architecture of the periodic βIV-spectrin scaffold at the axon initial segment with live electrophysiological patch-clamp recordings [71]. By mitigating depth-dependent aberrations and tissue scattering, the combination of AO-SRM and two-photon fluorescence microscopy provides a robust methodological framework for characterizing subcellular dynamics in vivo. The deep-tissue approach extends to non-invasive intravital investigations through highly distorting cranial barriers. Employing a dual-mode strategy that pairs direct hardware sensing with iterative sensorless methods, AO-driven two-photon imaging has resolved subcellular mitochondrial networks and tracked organelle dynamics directly through the intact skull of live mice [97]. This volumetric capability supports the evaluation of dynamic neuroglial interactions at the tripartite synapse, cellular organelle networks, and brain extracellular space anatomy through highly scattering structural barriers. Beyond rodent models, AO architectures have expanded the horizons of neurodevelopmental and translational research, enabling subcellular reconstructions within the complex circuits of the adult *Drosophila* brain [72] and deep imaging of hiPSC-derived neuronal axons under highly aberrated tissue environments [80]. Additionally, when augmented by computational approaches like Self-Net, these frameworks can handle the immense data demands required for the isotropic restoration of intricate neuronal morphologies within terabyte-scale whole-brain datasets [82]. In pathological contexts, AO-SRM provides a quantitative framework to analyze neurodegenerative mechanisms by visualizing how amyloid-beta and tau assemblies [70] disrupt subcellular structural integrity, receptor mobility, and downstream signaling cascades [98–102].

## Data Availability

No datasets were generated or analysed during the current study.

## Abbreviations

AO: Adaptive optics
AO-SRM: Adaptive optics super-resolution microscopy
CNN: Convolutional neural network
DL: Deep learning
DM: Deformable mirror
DNA-PAINT: DNA point accumulation for imaging in nanoscale topography
GAN: Generative adversarial network
LFM: Light field microscopy
LLSM: Lattice light sheet microscopy
LSM: Light sheet microscopy
PALM: Photo-activated localization microscopy
PSF: Point spread function
SH-WFS: Shack-Hartmann wavefront sensor
SIM: Structured illumination microscopy
SLM: Spatial light modulator
SMLM: Single-molecule localization microscopy
SRM: Super-resolution microscopy
SRRF: Super-resolution radial fluctuation
STED: Stimulated emission depletion
STORM: Stochastic optical reconstruction microscopy
